# Overexpression of miR-155 in the Liver of Transgenic Mice Alters the Expression Profiling of Hepatic Genes Associated with Lipid Metabolism

**DOI:** 10.1371/journal.pone.0118417

**Published:** 2015-03-23

**Authors:** Xiaolin Lin, Junshuang Jia, Tao Du, Wei Li, Xiaoyan Wang, Jieqiong Wei, Xia Lin, Hui Zeng, Longping Yao, Xuebing Chen, Jingshen Zhuang, Jie Weng, Yu Liu, Jihong Lin, Qinghong Wu, Wanshan Wang, Kaitai Yao, Kang Xu, Dong Xiao

**Affiliations:** 1 Cancer Research Institute, Southern Medical University, Guangzhou, China; 2 Institute of Comparative Medicine & Laboratory Animal Center, Southern Medical University, Guangzhou, China; 3 Department of General Surgery, Sun Yat-sen Memorial Hospital of Sun Yat-sen University, Guangzhou, China; 4 Department of Endocrinology, The Second Affiliated Hospital, Guangzhou Medical University, Guangzhou, China; 5 Department of Medical Imaging Center, Nanfang Hospital, Southern Medical University, Guangzhou, China; 6 Zhujiang Hospital, Southern Medical University, Guangzhou, China; 7 School of Traditional Chinese Medicine, Southern Medical University, Guangzhou, China; Northeast Ohio Medical University, UNITED STATES

## Abstract

Hepatic expression profiling has revealed miRNA changes in liver diseases, while hepatic miR-155 expression was increased in murine non-alcoholic fatty liver disease, suggesting that miR-155 might regulate the biological process of lipid metabolism. To illustrate the effects of miR-155 gain of function in transgenic mouse liver on lipid metabolism, transgenic mice (i.e., Rm155LG mice) for the conditional overexpression of mouse miR-155 transgene mediated by Cre/lox P system were firstly generated around the world in this study. Rm155LG mice were further crossed to Alb-Cre mice to realize the liver-specific overexpression of miR-155 transgene in Rm155LG/Alb-Cre double transgenic mice which showed the unaltered body weight, liver weight, epididymal fat pad weight and gross morphology and appearance of liver. Furthermore, liver-specific overexpression of miR-155 transgene resulted in significantly reduced levels of serum total cholesterol, triglycerides (TG) and high-density lipoprotein (HDL), as well as remarkably decreased contents of hepatic lipid, TG, HDL and free fatty acid in Rm155LG/Alb-Cre transgenic mice. More importantly, microarray data revealed a general downward trend in the expression profile of hepatic genes with functions typically associated with fatty acid, cholesterol and triglyceride metabolism, which is likely at least partially responsible for serum cholesterol and triglyceride lowering observed in Rm155LG/Alb-Cre mice. In this study, we demonstrated that hepatic overexpression of miR-155 alleviated nonalcoholic fatty liver induced by a high-fat diet. Additionally, carboxylesterase 3/triacylglycerol hydrolase (Ces3/TGH) was identified as a direct miR-155 target gene that is potentially responsible for the partial liver phenotypes observed in Rm155LG/Alb-Cre mice. Taken together, these data from miR-155 gain of function study suggest, for what we believe is the first time, the altered lipid metabolism and provide new insights into the metabolic state of the liver in Rm155LG/Alb-Cre mice.

## Introduction

MicroRNAs (miRNAs), a class of small non-coding RNA molecules, function by regulating gene expression via degradation or translational inhibition of their target mRNAs, and thus participate in a wide variety of physiological and pathological cellular processes including: development, cell proliferation, differentiation and apoptosis, metabolism, cancer and etc [1,2]. As a typical multifunctional miRNA, miR-155 plays a crucial role in various physiological and pathological processes, such as haematopoietic lineage differentiation, immunity, inflammation, cardiovascular diseases and cancer [3,4]. The available experimental evidence indicates that miR-155 is abnormally expressed in a variety of human tumor tissues, and has been found to be associated with cancer initiation, progression, metastasis and prognosis [3,4].

On the other hand, there are several lines of evidence that miR-155 is involved in adipocyte differentiation, adipogenesis and obese [5–8], indicating that it might play a significant role in the process of lipid metabolism. In subcutaneous adipose tissue, miR-155 was significantly higher expression in normal glucose tolerance group as compared to the type 2 diabetes group [5]. In vitro, TNF-α treatment resulted in the up-regulation of miR-155 and this overexpression of miR-155 inhibited adipogenesis by down-regulating early adipogenic transcription factors [6]. During the adipogenic program of both immortalized and primary hMSCs, the expression of miR-155, miR-221, and miR-222 decreased, however, ectopic expression of these miRNAs significantly inhibited adipogenesis [7]. In vivo, overexpression of miR-155 in transgenic mice causes the reduction of brown adipose tissue mass and impairment of brown adipose tissue function [8]. In contrast, inhibition of miR-155 enhances brown adipocyte differentiation and induces a brown adipocyte-like phenotype ('browning') in white adipocytes [8]. In addition, hepatic miR-155 expression was increased in murine non-alcoholic fatty liver diseases (NAFLD) [9,10], and miR-155 might play a protective role in the development of non-alcoholic hepatosteatosis in mice [10]. Moreover, miR-155 negatively regulates lipid uptake in oxLDL(oxidized low-density lipoprotein)-stimulated dendritic cells/macrophages [11]. The aforementioned findings suggest that hepatic miR-155 expression might regulate the biological processes of lipid metabolism, which remains to be fully characterized.

Against the background, transgenic mice (i.e., Rm155LG mice) for the conditional overexpression of mouse miR-155 transgene mediated by Cre/lox P switching expression system were successfully generated in this study, while Rm155LG mice were further crossed to Alb-Cre mice to realize the liver-specific overexpression of mouse miR-155 transgene in Rm155LG/Alb-Cre double transgenic mice, which will be employed to explore the effects of the overexpression of miR-155 in the transgenic mouse livers on the expression profiling of hepatic genes associated with lipid metabolism, and on blood and hepatic lipid contents.

## Materials and Methods

### 1. Production of the Rm155LG transgenic mice

A 318bp fragment containing the precursor sequence of the mmu-miR-155 was amplified by PCR from pEμ-mmu-miR155 plasmid [12], and then directionally cloned into the *Mlu* I and *Sac* I sites of the pRLG plasmid [13,14], designated as pRm155LG, followed by identification of PCR, enzyme digestion analysis and sequencing (data not shown).

**Fig 1:**
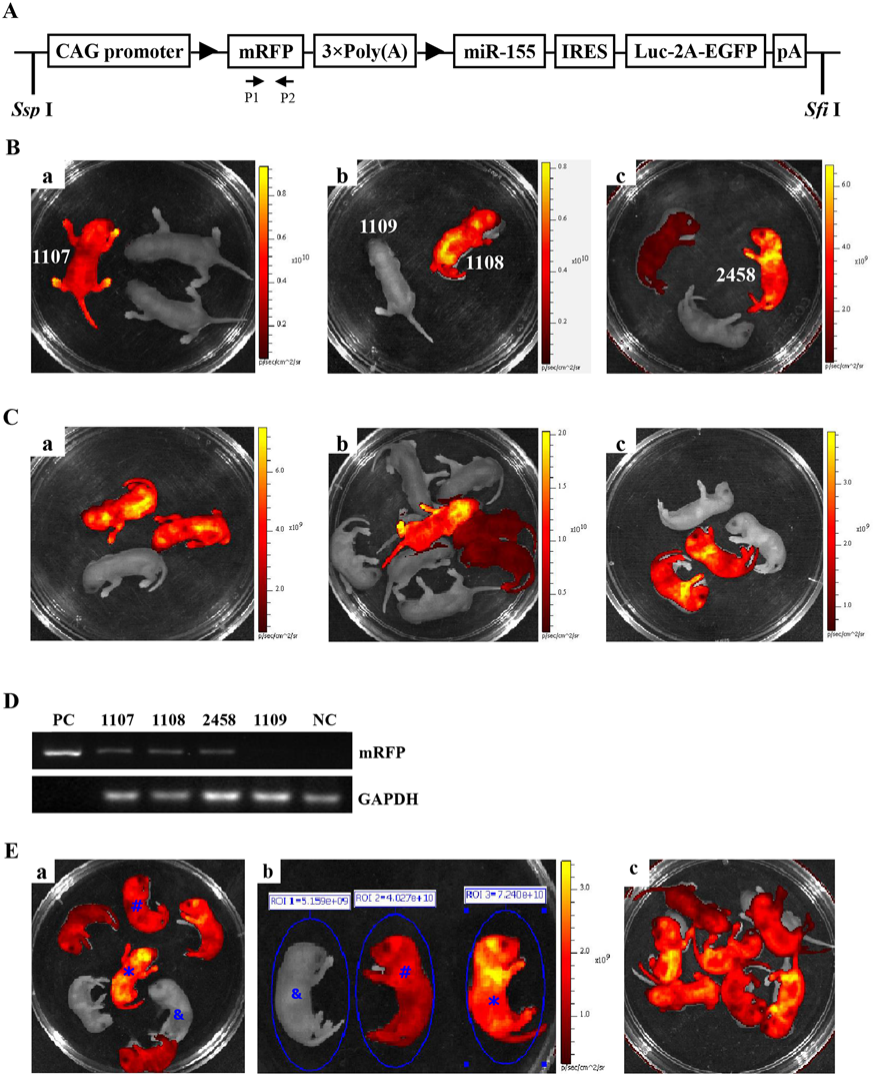
Generation of Rm155LG transgenic mice. (**A**) Schematic diagram of the Rm155LG transgenic construct used to generate Rm155LG transgenic mice. A potent, ubiquitous CMV/β-actin promoter in the vector pRm155LG was used to drive a series of cassettes, including a floxed mRFP followed by a triple transcription-stopping polyA sequence (3×PolyA) and a downstream internal ribosome entry site (IRES)-based bicistronic transcript, including open-reading frames of mouse miR-155 and a multifunctional marker consisting of firefly Luc fused to eGFP with a transmembrane-localizing domain (Luc-TMeGFP). The primer pair P1/P2 represented by small arrows were used in PCR analysis of genotype to detect reporter transgene mRFP. Only mRFP will be transcribed and expressed properly from this construct, while Cre-mediated recombination occurs, the floxed mRFP+3×PolyA is excised, and the downstream, bicistronic transcript is activated. The multifunctional marker will be expressed, replacing mRFP in Cre-activated cells. The construct map is not drawn to the scale. **Abbreviations**: **CAG promoter**: CMV early enhancer/chicken β actin promoter; **mRFP:** monomeric red fluorescent protein; **Luc**: firefly luciferase; **EGFP**: enhanced green fluorescent protein; **pA**: polyadenylation signal; **The black triangle**: lox P site. **(B)** Screening Rm155LG transgenic founders by *in vivo* non-invasive fluorescence imaging. Three foster mothers gave birth to three, two and three F0 pups, respectively; three mRFP-positive Rm155LG transgenic mice (referred to as 1107^#^, 1108^#^ and 2458^#^) with strong red fluorescence were found via mRFP assay by using the Xenogen IVIS Lumina Imaging System 2–3 days after birth. **(C)** F1 progeny inherit and express mRFP transgene from three founders. Offspring shown in Fig. 1C-a,b,c were derived from the mating between founder 1107^#^, 1108^#^ or 2458^#^ and wildtype mouse, respectively. A fraction of founder offspring with mRFP fluorescence showed that all of three founders could transmit Rm155LG transgene to subsequent generation (i.e., F1). **(D)** mRFP-positive founders verified for Rm155LG transgene presence by PCR analysis. Three mRFP-positive mice (i.e., 1107^#^, 1108^#^ or 2458^#^) and one mRFP-negative mice (i.e., 1109^#^) were individually analyzed by PCR for the genomic integration of transgene with tail biopsy-derived DNA from mice (1107^#^, 1108^#^, 1109^#^ and 2458^#^). PCR products were amplified by the primer pair P1/P2 (specific for mRFP) shown in Fig. 1A. **lane PC**: positive control (pRm155LG as template); **lane NC**: negative control using genomic DNA from WT mouse as template. Data are representative of three independent PCR experiments that yield similar results. **(E)** Rapidly and readily distinguishing homozygous from heterozygous Rm155LG transgenic alleles by in vivo fluorescence imaging.

Rm155LG transgenic mice were generated by microinjection of DNA into the pronuclei of fertilized single-cell mouse embryos using standard techniques as previously described [15,16]. The C57BL/6 mouse strain, supplied by Laboratory Animal Center, Southern Medical University, was used as the source of embryos for the micromanipulation and for subsequent breeding trials. For microinjection, the fragment of Rm155LG transgene (Fig. 1A) was released free from the vector backbone of pRm155LG via digestion with *Ssp* I and *Sfi* I. Rm155LG transgenic mice from potential transgenic founders were preliminarily screened via mRFP assay by the Xenogen IVIS Lumina Imaging System 2–3 days after birth, and subsequently confirming the results of mRFP assay by PCR-based genotyping performed on tail-extracted genomic DNA.

Homozygous animals of Rm155LG transgenic mouse lines were obtained by intercrosses of Rm155LG heterozygotes derived from mating between Rm155LG transgenic founder (1107^#^, ♀) and wild-type C57BL/6 mouse strain (♂), followed by optically differentiating homozygous Rm155LG transgenic mice by in vivo (whole-body, newborn) qualitative (Fig. 1E-a) and quantitative (Fig. 1E-b) fluorescence imaging, which was further confirmed by mouse mating (Fig. 1E-c).

This study was carried out in strict accordance with the recommendations in the Guide for the Care and Use of Laboratory Animals of the Southern Medical University. The protocol was approved by the Committee on the Ethics of Animal Experiments of the Southern Medical University. All surgery was performed under sodium pentobarbital anesthesia, all efforts were made to minimize animal suffering and the number of animals used was kept to a minimum by the experimental design.

### 2. Whole-animal (in vivo) and organ (ex vivo) fluorescence imaging

The procedure for whole-animal and organ mRFP (monomeric red fluorescent protein) fluorescence imaging via using stereo fluorescence microscope (Nikon, AZ100) or the Xenogen IVIS Lumina II Imaging System was previously fully described [15].

### 3. Genotype analysis by PCR

PCR was performed on tail genomic DNA to further identify Rm155LG integrated into their genome. The sequences of the forward primer (FP) and reverse primer (RP) used to amplify a 339-bp fragment of the Rm155LG transgene were: 5’-GGGAGCGCGTGATGAAC-3’ (FP) and 5'-CGTTGTGGGAGGTGATGTC-3' (RP). PCR conditions were as follows: pre-denaturation at 94°C for 7 min, followed by 30 amplification cycles of denaturation at 94°C for 1 min, primer annealing at 54°C for 1 min, and extension at 72°C for 30 s, and finally an additional extension at 72°C for 10 min. Rm155LG construct DNA was used as the positive control for each PCR reaction, and genomic DNA from wildtype mice was employed as a negative control for each PCR test.

### 4. Establishment of homozygous Rm155LG transgenic mouse colonies by in vivo fluorescence imaging

Procedure for rapidly and readily distinguishing homozygous Rm155LG transgenic mice from F2 generation derived from Rm155LG transgenic founder by in vivo qualitative and quantitative fluorescence imaging immediately after birth via using the IVIS Lumina II imaging system (Xenogen Corp., Alameda, CA) was detailedly illustrated in our publication [15].

### 5. In vivo and ex vivo optical imaging of firefly luciferase (Luc) activity

Rm155LG mice were crossed to homozygous Alb-Cre mice (B6.Cg-Tg(Alb-cre)21Mgn/J) (obtained from Model Animal Research Center of Nanjing University) to generate Rm155LG/Alb-Cre double transgenic mice, in which Luc expression was activated in liver-restricted pattern, as determined by the non-invasive in vivo bioluminescence imaging. Bioluminescence was measured non-invasively using the IVIS Lumina II imaging system. The protocols for whole-animal (in vivo) and dissected organ (ex vivo) bioluminescence imaging to detect Luc activity by the IVIS system were previously well described [15].

### 6. RNA isolation and quantitative real-time PCR (qRT-PCR)

To quantitate miRNA and mRNA expression, total RNA was extracted from the liver of Rm155LG/Alb-Cre transgenic mice and control mice with the use of TRIzol reagent (TaKaRa). Total RNA was reversely transcribed with the PrimeScript RT reagent Kit (TaKaRa). qRT-PCR was performed using SYBR Green PCR master mix (TaKaRa) on Stratagene Mx3005P qRT-PCR System according to the manufacturer’s instructions. The primers used for the amplification of the indicated genes were listed in S1–S3 Tables. To measure the levels of miR-155 and the indicated mRNAs, U6 snRNA and GAPDH were used as endogenous control, respectively.

### 7. mRNA microarray analysis

Expression microarray analysis was carried out with commercially available 32K mouse genome array (CapitalBio Corp., Bejing, China). Total RNA samples were extracted from the liver of Rm155LG/Alb-Cre transgenic mice and control mice using TRIzol reagent (TaKaRa). All the hybridization procedures and data analysis were performed by CapitalBio Corp. (Bejing, China). Briefly, total RNA was used to synthesize cDNA in an in vitro transcription reaction, and then cDNA was fluorescently labeled by Cy5-dCTP or Cy3-dCTP (GE Healthcare Cat. No. PA 55021/PA 53021) with Klenow enzyme. Labeled cDNA was then hybridized to 32K mouse genome arrays. Hybridization signals were scanned with a Lux-Scan 3.0 scanner (CapitalBio Corp., Bejing, China). The resultant images were digitized with Lux-Scan 3.0 image analyzer software (CapitalBio Corp., Bejing, China). The microarray data were deposited in a database (ArrayExpress, GEO) with accession number GSE64255.

### 8. Morphometric and histochemical analysis of livers

The mice were killed after an overnight fast. Livers from Rm155LG/Alb-Cre transgenic mice and control mice were fixed in 10% formalin for at least 24 hours or kept freshly frozen. Formalin-fixed, paraffin-embedded liver sections (5μm) were stained with hematoxylin and eosin (H&E) for morphologic studies under light microscope. Oil red O (ORO) staining was performed on frozen liver sections (10μm).

### 9. Assay of blood and hepatic lipids

The hepatic lipids were extracted from liver tissue using chloroform/methanol mixed solution (1:1, vol:vol), the prepared sample was then centrifuged at 1200 g for 10 min, and then the obtained supernatant was used for lipid measurements. Triglycerides (TG) were measured by the glycerol phosphate oxidase-peroxidase method, Total cholesterols (TC) by the cholesterol oxidase-peroxidase method, high-density lipoprotein cholesterol (HDL-C) by the direct method-surfactant clearance method, low-density lipoprotein cholesterol (LDL-C) by the direct method-selected inhibitor method, free fatty acid (FFA) by the Wako enzymatic method, alanine transaminase (ALT) by the IFCC Reference method with P-5-P, and aspartate aminotransferase (AST) by the IFCC Reference method with P-5-P, using TBA-120 auto-analyzer (Toshiba Medical Systems, Japan), respectively.

### 10. High-fat diet (HFD) experiments

In high-fat diet experiments, Rm155LG/Alb-Cre mice and control mice were fed normal chow diet or HFD (60% energy from fat, Research Diets D12492) for 6 months until sacrifice. At the end of the experiment, mice were fasted for 12h, euthanized, and body weights were determined. Blood samples were collected immediately and serum was obtained by centrifugation at 3000rpm for 15 min at 4°C. Livers were removed, weighed, and snap-frozen in liquid nitrogen. Liver and serum samples were stored at -80°C until RNA isolation or biochemical analysis.

### 11. Luciferase reporter assay

The dual luciferase reporter gene plasmid (i.e., pLuc-Ces3–3’-UTR-wt) containing the putative miR-155 binding site at the 3’-UTR of Ces3 mRNA was purchased from Kangbio (Shenzhen, China). Cells were seeded in 48-well plates and cultured for 48 hours. The pLuc- Ces3–3’-UTR-wt plasmid was co-transfected into mouse NIH3T3 cells with the miR-155 mimics, mimics control, miR-155 inhibitor or inhibitor control using Lipofectamine 2000 Reagent (Invitrogen), respectively. Luciferase and Renilla activities were assayed 48 hours after transfection using the Dual Luciferase Reporter Assay Kit (Promega) following the manufacturer’s instructions.

## Results

### 1. Generation of Rm155LG transgenic mice

To realize the above-mentioned purposes and attain further insight into the cell/tissue-specific and/or developmental stage-specific roles of miR-155 in vivo, we want to produce transgenic mice that could conditionally overexpress mouse miR-155 transgene mediated by Cre/lox P system (Fig. 1A).

The Rm155LG construct used for microinjection was illustrated in Fig. 1A. mRFP expression allows rapid and easy identification of Rm155LG transgenic mice by mRFP assay. Of the 396 embryos transferred to the recipient females, 22 embryos developed to term. Three individuals (with strong red fluorescence) of 22 siblings were transgenic, as demonstrated by the red fluorescence in the whole body of newborn mice (Fig. 1B), as confirmed by PCR analysis (Fig. 1D). Thus, we gained three founder animals (referred to as 1107^#^, 1108^#^ and 2458^#^) that expressed strongly mRFP and were normal in phenotype.

In addition, to determine whether the Rm155LG transgene was passaged to the next generation, founders (i.e., referred to as 1107^#^, 1108^#^ and 2458^#^) was back-crossed to the parental wildtype strain to give F1 generation, respectively. Fig. 1C demonstrated that all of three founders could transmit the Rm155LG foreign transgene to subsequent generation (i.e., F1) since a fraction of founder offspring showed mRFP fluorescence.

### 2. mRFP visualization in postnatal organs of Rm155LG transgenic mice

We next determined the expression pattern of the mRFP transgene in organs taken from Rm155LG transgenic mice. Red fluorescence was detected in tissue/organ samples including heart, liver, spleen, lung, kidney, stomach, brain, testis, intestine, muscle, thymus, skin, eye and pancreas isolated from the transgenic positive mice, but not in control littermates (S1A Fig.). Additionally, the brain, pancreas, muscle, testis, stomach and skin showed strong red fluorescence, while the heart, liver, lung, kidney, intestine, spleen, thymus, eye and fat demonstrated relatively weaker fluorescence signals (S1B Fig.). In summary, all tissues exhibited mRFP expression, indicating ubiquitous expression of the transgenic cassette. In general, Rm155LG transgenic mice were viable and fertile, and manifested no gross behavioral or phenotypic abnormalities.

### 3. Visually identifying homozygous Rm155LG transgenic mice by whole-body fluorescence imaging of newborn offspring

Rm155LG transgenic founder 1107^#^ (♀) selected from above-mentioned three founder animals (Fig. 1B) were employed to fully demonstrate how to simply, rapidly and visually distinguish homozygous from heterozygous animals by whole-body (newborn) fluorescence imaging. Procedure for establishing homozygous Rm155LG transgenic mouse colony by mating heterozygous males and females from founder line 1107^#^ was detailedly demonstrated in “MATERIALS AND METHODS” and the legend of Fig. 1E. Brother sister mating of mRFP-positive heterozygous animals (Fig. 1C-a) showed transgene transmission to the offspring (5 mRFP-positive and 2 mRFP-negative) following expected Mendelian laws (Fig. 1E-a). The in vivo qualitative imaging data greatly facilitated us to rapidly and readily find 5 mRFP-positive mice out of 7 littermates; among 5 mRFP-positive mice, one mRFP-positive transgenic mice [marked with asterisk (*)] indicated more strong red fluorescence (Fig. 1E-a) and very high fluorescence intensity (FI) (Fig. 1E-b) in whole body (newborn), compared with the rest of 4 mRFP-positive littermates. Therefore, we thought that one mRFP-positive transgenic mouse marked by asterisk (*) was regarded as homozygous for Rm155LG transgene based on the qualitative and quantitative data from in vivo fluorescence imaging, as strongly supported by mouse mating (Fig. 1E-c). Moreover, the reliability and repeatability of this optical assay for screening homozygous Rm155LG transgenic mice used in this study were strongly supported by our findings [15] and other finding [17,18]. Additionally, this optical approach greatly allowed us to easily and rapidly obtain homozygous Rm155LG transgenic mouse colonies derived from other Rm155LG transgenic founders (i.e., 1108^#^ and 2458^#^)] (data not shown). In summary, the in vivo fluorescence imaging is valuable as a visual, rapid, reliable and accurate screening tool for homozygous transgenic mice (harboring fluorescence reporter transgene under the control of a ubiquitous promoter) immediately after birth, as supported by this study and other findings [15,17,18].

### 4. Liver-specific overexpression of miR-155 transgene in transgenic mice mediated by Cre/lox P system

**Fig 2:**
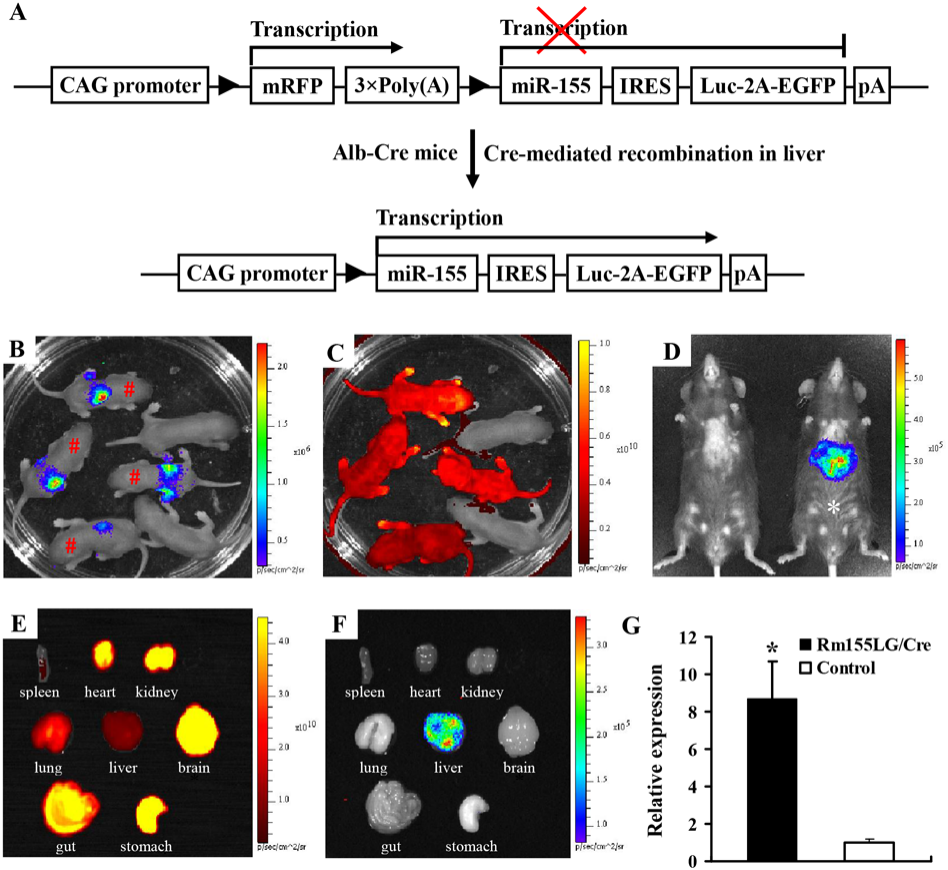
Liver-specific overexpression of mouse miR-155 in transgenic mice mediated by Cre/*lox* P system. (**A**) Strategy for liver-specific expression of miR-155 transgene using Cre/*lox* P system. In the absence of Cre-mediated recombination, only mRFP will be transcribed, while miR-155 and Luc transgene expression is prevented by STOP sequence flanked by *lox* P sites. When Cre-mediated recombination occurs in mouse liver, the floxed mRFP + 3×PolyA is excised, and miR-155 and Luc transgene expression is activated in a liver-restricted pattern in Rm155LG/Alb-Cre double transgenic mice. Other details as in Fig. 1A. **(B–C)** Whole-body bioluminescence (B) and fluorescence (C) imaging for newborn offspring derived from mating heterozygous Rm155LG transgenic mice with homozygous Alb-Cre mice. (**D**) In vivo luciferase imaging in the liver of both adult Rm155LG/Alb-Cre mouse and the control littermate developing from these offspring shown in Fig. 2B-C. (**E-F**) Ex vivo imaging of mRFP (E) and luc (F) expression in organs from same mouse shown [marked by asterisk (*)] in Fig. 2D. (**G**) qRT-PCR for mouse miR-155 transgene expression in liver from double transgenic mouse (Rm155LG/Alb-Cre^Tg^) and littermate control. *, P < 0.05 compared with control.

Next, we confirmed that the expression of miR-155 transgene in Rm155LG transgenic mice could be induced in a Cre-dependent manner. To analyze the expression of miR-155 transgene in mouse liver, we crossed heterozygous Rm155LG transgenic mice with homozygous Alb-Cre mice in which Cre is under the control of the liver-specific albumin (Alb) promoter [19] to generate Rm155LG/Alb-Cre double transgenic mice in which miR-155 and Luc transgene expression was expected to be activated in liver-restricted manner (Fig. 2A), as determined by qRT-PCR and the noninvasive in vivo bioluminescence imaging, respectively. Whole-animal bioluminescence imaging indicated that Luc activity in the liver of Rm155LG/Alb-Cre transgenic mice could be detected in mRFP-positive newborn offspring (Fig. 2B, C) and adult mouse (Fig. 2D), suggesting the successful activation of Luc expression mediated by Alb-Cre. Organ-specific bioluminescence imaging showed that Luc activity could be assayed in liver, but not in other organs (Fig. 2F) obtained from Luc-positive mouse (genotype: Rm155LG/Alb-Cre) [marked by asterisk (*)] shown in Fig. 2D, while Luc activity could not be detected in all of organs [mentioned in (Fig. 2E-F)] obtained from Luc-negative mouse shown in Fig. 2D (data not shown). qRT-PCR using RNA extracted from the liver of Luc-positive and Luc-negative mice (shown in Fig. 2D) exhibited a significant increase in the levels of miR-155 transgene (Fig. 2G). These data lead us to conclude that the Rm155LG conditional transgenic system worked in a Cre-dependent manner.

### 5. Liver-specific overexpression of miR-155 transgene resulted in altered hepatic and serum lipid profiles

**Fig 3:**
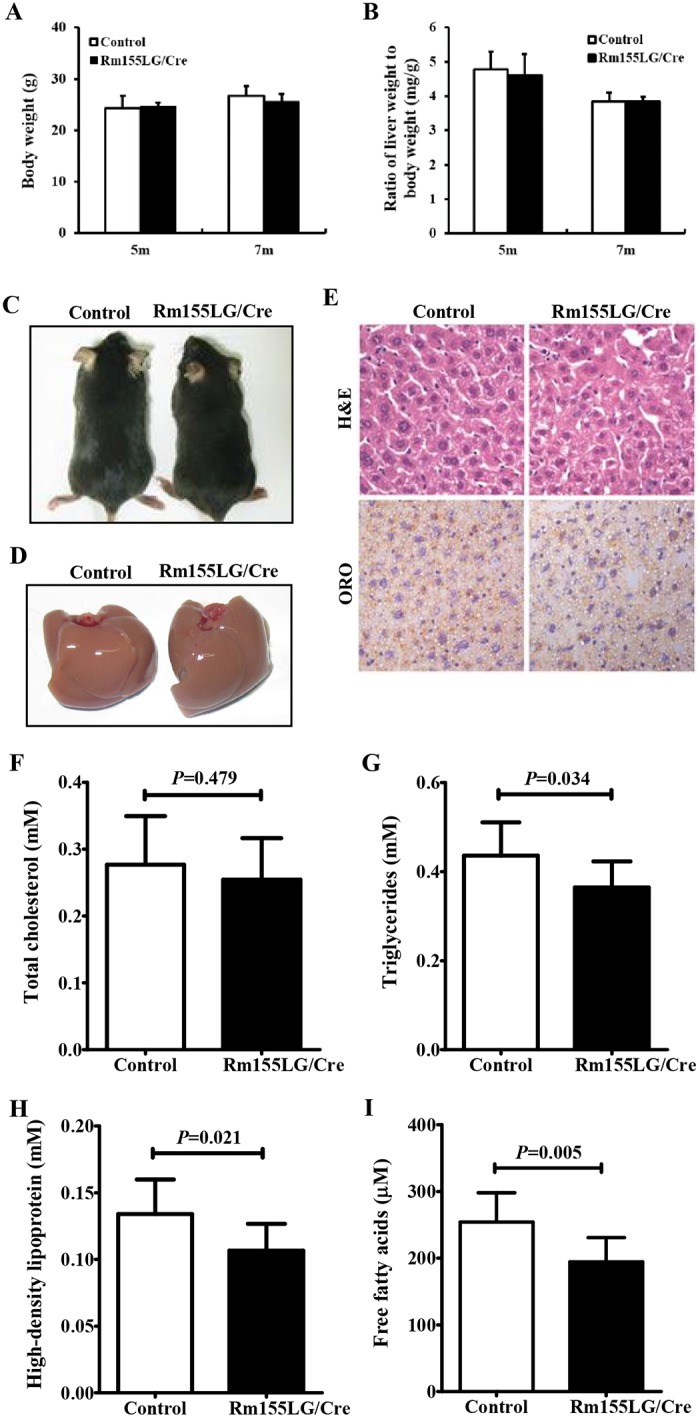
Rm155LG/Alb-Cre mice improved lipid metabolism in liver. (**A**) Body weight of Rm155LG/Alb-Cre mice and controls at different ages. (**B**) Relative liver weight of Rm155LG/Alb-Cre mice vs. controls. (**C**) Adult Rm155LG/Alb-Cre mouse (right) and control (left) fed a normal diet. (**D**) Gross morphology of Rm155LG/Alb-Cre mouse (right) and control (left) livers. **(E)** H&E staining and Oil red O (ORO) staining of liver sections from control and Rm155LG/Alb-Cre mice. (**F**) Quantification of TC, TG, HDL and FFA storage in the liver of control and Rm155LG/Alb-Cre mice. TC, total cholesterols; TG, triglycerides; HDL, high-density lipoprotein; FFA, free fatty acids. Data are mean±SD (n = 9–10). Statistical significance was determined by two-tailed student t-test.

The enforced expression of miR-155 in the liver of Rm155LG/Alb-Cre mice did not alter the final body weight and liver weight of Rm155LG/Alb-Cre mice at different ages (Fig. 3A, B, C and Table 1). No evident difference in gross morphology and appearance of livers was found between control and Rm155LG/Alb-Cre mice (Fig. 3D). Moreover, the weight of epididymal fat pads did not differ between groups (Table 1).

**Table 1:** Blood profile for control and Rm155LG/Alb-Cre mice.

Parameters	Control (n = 11)	Rm155LG/Cre (n = 9)
Body weight (g)	19.72±1.65	19.59±1.05
Liver (g)	1.18±0.09	1.13±0.08
WAT (g)	0.25±0.04	0.22±0.06
TC (mmol/l)	2.04±0.23	1.76±0.22*
TG (mmol/l)	1.31±0.29	0.96±0.14*
LDL (mmol/l)	0.50±0.06	0.50±0.08
HDL (mmol/l)	1.69±0.24	1.33±0.24*
AST (U/L)	111.6±14.7	125.0±23.4
ALT (U/L)	62.5±7.2	68.7±13.7

Cre control mice were littermates of the Rm155LG/Alb-Cre mice. Aspartate aminotransferase (AST), alanine transaminase (ALT), cholesterol and triglyceride values were determined in serum. WAT, white adipose tissue; TC, total cholesterol; TG, triglycerides; LDL, low-density lipoprotein; HDL, high-density lipoprotein. Data are mean ± SD. Statistical significance was determined by two-tailed student t-test.

*, *P*<0.05.

We next examined liver function in 2-month-old Rm155LG/Alb-Cre mice by measuring the serum profile of liver enzymes and metabolites after overnight fasting (Table 1). As shown in Table 1, no significant differences were found in serum AST, ALT or LDL, but significant decreases in serum concentrations of TC (≈12%, P = 0.014), TG (≈31%, P = 0.004) and HDL (≈22%, P = 0.005) were observed in Rm155LG/Alb-Cre mice when compared with control mice.

These reduced changes in blood TC, TG and HDL contents found in Rm155LG/Alb-Cre mice prompted us to further measure hepatic lipid parameters of Rm155LG/Alb-Cre mice and control mice (Fig. 3E-I). Staining mouse livers with Oil Red O (ORO) demonstrated decreased lipid deposition in Rm155LG/Alb-Cre mice compared with control mice (Fig. 3E). A detailed analysis of the lipid composition revealed that liver TG (≈16%, P = 0.034) (Fig. 3G), HDL (≈20%, P = 0.021) (Fig. 3H) and FFA (≈23%, P = 0.005) (Fig. 3I) levels were significantly decreased in livers of Rm155LG/Alb-Cre mice vs control mice, but hepatic TC (Fig. 3F) and LDL (data not shown) levels did not significantly differ between control and Rm155LG/Alb-Cre mice. Thus, liver-specific overexpression of miR-155 transgene leads to the altered hepatic and serum lipid profiles.

### 6. Transcriptional profile revealed altered expression pattern of hepatic lipid metabolism genes in Rm155LG/Alb-Cre mouse liver

To investigate the mechanisms underlying the abnormalities observed in Rm155LG/Alb-Cre mice, we further examined hepatic gene expression in control and Rm155LG/Alb-Cre mice (*n* = 3 of each genotype) by microarray analysis. A total of 168 and 470 genes were significantly upregulated and downregulated, respectively, in Rm155LG/Alb-Cre mouse livers compared with the control livers (S2 Fig. and S4 Table).

**Fig 4:**
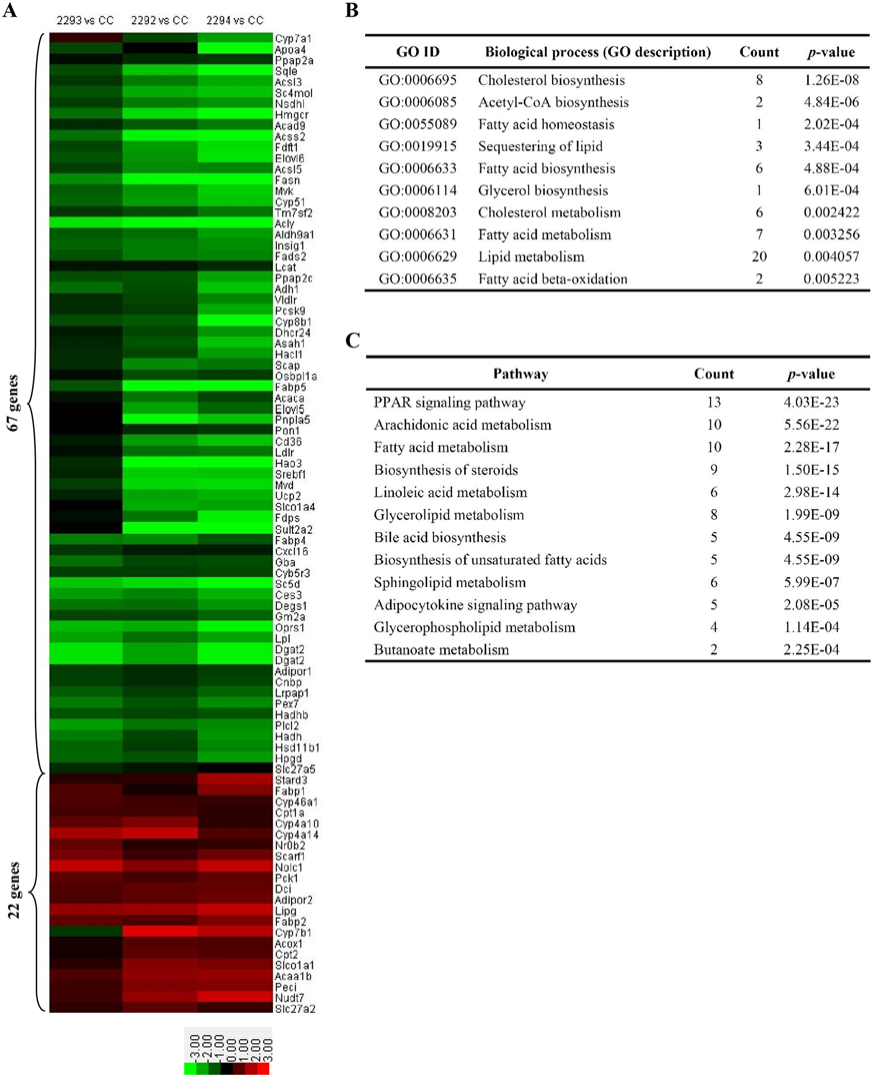
Microarray revealed the altered hepatic lipid metabolism genes in the liver of Rm155LG/Alb-Cre mice. (**A**) Class comparison and hierarchical clustering of differentially expressed hepatic lipid metabolism-related genes between Rm155LG/Alb-Cre and control mouse liver. A cluster heat map for hepatic lipid metabolism-related genes (see S6 Table, S7 Table and S8 Table) is shown. Other details as in S2 Fig. (**B-C**) Gene ontology (GO) (B) and KEGG pathway (C) analyses of up- and down-regulated genes between Rm155LG/Alb-Cre and control mouse liver. Genes with expression changes of greater than 2-fold with P values below 0.05 were identified and classified using GO categories.

**Fig 5:**
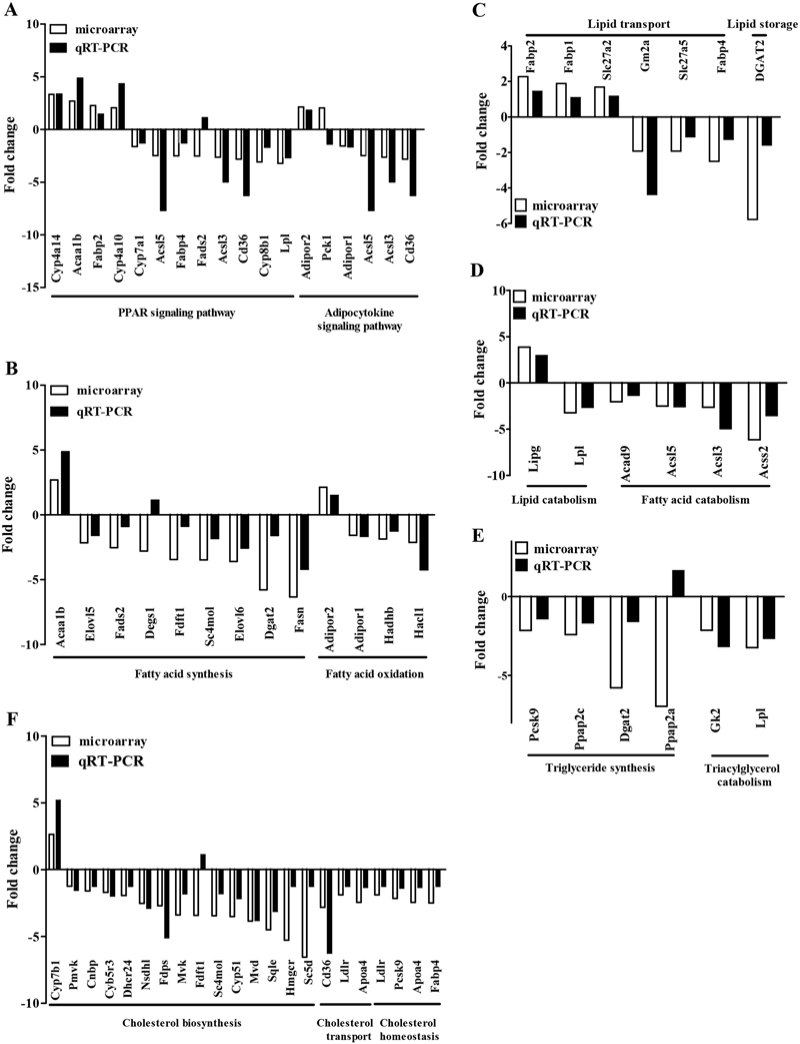
cDNA microarray and qRT-PCR revealed a general downward trend in the expression of hepatic cholesterol, triacylglycerol and fatty acid synthesis-related genes in Rm155LG/Alb-Cre transgenic mouse liver. Graph illustrating the fold change in gene expression of representative differentially hepatic lipid metabolism-related genes between Rm155LG/Alb-Cre and control mouse liver. qRT-PCR validated microarray-derived data on the increased or decreased mRNA expression of hepatic lipid metabolism-related genes in Rm155LG/Alb-Cre transgenic mouse liver. Additionally, a cluster heat map for hepatic lipid metabolism-related genes (see S6 Table and S7 Table) is shown in Fig. 4A. Other details as in S2 Fig.

The genes with significant express changes (S2 Fig.) were submitted to the DAVID online tool (http://david.abcc.ncifcrf.gov/home.jsp) for gene ontology (GO) annotation and pathway enrichment analysis (Fig. 4B, C and S8 Table). As we expected, GO analysis of the 638 genes displaying significant changes in the expression of Rm155LG/Alb-Cre mouse liver illustrated a significant enrichment for 89 genes (up-regulated: 22; down-regulated: 67) (Fig. 4A and S5–S7 Tables) with functions typically associated with lipid metabolism (e.g., lipid metabolism, sequestering of lipid, cholesterol metabolism, cholesterol biosynthesis, acetyl-CoA biosynthesis, glycerol biosynthesis, fatty acid metabolism, fatty acid biosynthesis, fatty acid beta-oxidation and fatty acid homeostasis) (Fig. 4B and S8 Table). Functional classification of the differentially expressed mRNA transcripts based on KEGG pathway analysis also demonstrated that the upregulated and downregulated genes in Rm155LG/Alb-Cre mouse livers are highly associated with PPAR signaling pathway, adipocytokine signaling pathway, fatty acid metabolism, biosynthesis of unsaturated fatty acids, bile acid biosynthesis, arachidonic acid metabolism, biosynthesis of steroids, linoleic acid metabolism, glycerolipid metabolism, sphingolipid metabolism, glycerophospholipid metabolism and butanoate metabolism (Fig. 4C and S8 Table). Additionally, qRT-PCR was used to validate the expression changes in transcript levels for a selected subset of hepatic lipid metabolism genes identified by microarray (Fig. 5). Together, these results from GO annotation and pathway enrichment analysis of 638 differentially expressed genes demonstrate a significant enrichment for 89 genes with functions typically associated with fatty acid, cholesterol and triacylglycerol metabolism (Fig. 4, Fig. 5 and S5–S8 Tables), indicating that these altered hepatic lipid metabolism genes could be responsible, or contribute to the altered hepatic and serum lipid profiles observed in Rm155LG/Cre transgenic mice.

### 7. cDNA microarray and qRT-PCR revealed the mostly decreased expression of hepatic fatty acid, cholesterol and triacylglycerol synthesis-related genes in Rm155LG/Alb-Cre transgenic mouse liver

The low serum TC, TG and HDL levels in Rm155LG/Alb-Cre transgenic mice warranted an in-depth analysis of hepatic lipid metabolism in these mice. We analyzed the expression of various hepatic genes involved in lipid metabolism using cDNA microarray and qRT-PCR. Interestingly, we observed a general downward trend in the expression of hepatic genes involved in lipogenesis, lipid transport, lipid storage, bile acid biosynthesis, fatty acid synthesis, fatty acid oxidation, fatty acid catabolism, cholesterol biosynthesis, cholesterol transport, cholesterol homeostasis and triglyceride synthesis in Rm155LG/Alb-Cre transgenic mice, compared with control littermates (Fig. 4A, Fig. 5 and S6–S8 Tables).

As shown in Fig. 4A, Fig. 5B, D, S6 Table and S8 Table, liver-specific overexpression of mouse miR-155 transgene led to the generally decreased expression of hepatic genes involved in fatty acid synthesis (Acly, Fasn, Dgat2, Elovl5, Elovl6, Sc4mol, Fdftl and Fads2), fatty acid oxidation (Ucp2, Pex7, Hacl1, Hadhb and Adipor1) and fatty acid catabolism (Acly, Acss2, Acsl3, Acsl5 and Acad9).

To help elucidate the mechanism of serum cholesterol and triglyceride lowering (Table 1) caused by liver-specific overexpression of miR-155 transgene in vivo, cDNA microarray and qRT-PCR experiments were performed. Interestingly, our microarray and qRT-PCR data revealed a trend toward reduction of the expression profile of hepatic genes associated with cholesterol and triglyceride metabolism in Rm155LG/Alb-Cre transgenic mouse livers, including cholesterol biosynthesis (e.g., Mvk, Mvd, Sc5d, Hmgcr, Sqle, Cnbp, Dhcr24, Nsdhl, Fdps, Sc4mol, Fdft1 and Tm7sf2), cholesterol transport and uptake (e.g., Cd36, Apoa4 and Ldlr), cholesterol homeostasis (e.g., Fabp4, Apoa4, Pcsk9 and Ldlr), triglyceride synthesis (Ces3, Ppap2a, Dgat2, Ppap2c and Pcsk9) and triacylglycerol catabolism (Lpl and Gk2) (Fig. 4A, Fig. 5E, F and S7–S8 Tables), indicating that the decreased expression of these hepatic genes involved in cholesterol and triglyceride metabolism might be responsible, or contribute to the decreased cholesterol and triglyceride levels observed in Rm155LG/Alb-Cre transgenic mice.

Collectively, liver-specific overexpression of miR-155 transgene in transgenic mice results in a general downward trend in the expression profile of hepatic genes involved in fatty acid, cholesterol and triglyceride metabolism, which is likely at least partially responsible for the serum cholesterol and triglyceride lowering observed in these mice.

### 8. Genes involved in retinol metabolism and hepatic drug metabolism

Our transcriptional profiling also showed significant gene-expression changes in genes involved in retinol metabolism, including upregulated genes (i.e., Retsat, Ugt2b1, Cyp4a14, Aldh1a1 and Cyp4a10) and downregulated genes (i.e., Rdh11, Adh1, Cyp2c50, Cyp2a12, Cyp3a41, Cyp2c38, Cyp3a11, Ugt1a6a, Dgat2, Ugt3a2, Ugt2b37, Cyp2b10, Cyp3a44, Cyp2a5, Cyp2b9 and Cyp2c40) in the liver of Rm155LG/Alb-Cre transgenic mice (S3 Fig. and S9 Table). Furthermore, we also found the remarkable changes in the mRNA levels of hepatic drug metabolizing enzyme genes (upregulated genes: Cyp2d9, Gsta2, Ugt2b1, Gstp1, Gstp2, Cyp2e1 and Aox1; downregulated genes: Cyp2c39, Adh1, Cyp2c50, Cyp2a12, Cyp2c38, Cyp3a11, Ugt1a6a, Cyp2b10, Cyp3a44, Fmo3, Cyp2a5, Cyp2b9, Cyp2f2, Cyp2c40, Tk1, Upb1, Dpyd, Upp2, Es1 and Ces3) (S3 Fig. and S9 Table) in the liver of Rm155LG/Alb-Cre transgenic mice, while the GO terms representing biological processes related to drug metabolism—cytochrome P450, metabolism of xenobiotics by cytochrome P450 and drug metabolism—other enzymes were listed in S10 Table.

In addition to genes that regulate lipid metabolism, vitamin metabolism and hepatic drug metabolism, cDNA microarray highlighted the abnormal expression of many genes involved in amino acid metabolism, nucleic acid metabolism, hormone metabolism and terpenoid biosynthesis (S4 Fig. and S9–S10 Tables), and glucose metabolism, cellular proliferation and cancer (illustrated in our upcoming publications) in the liver of Rm155LG/Alb-Cre transgenic mice. In summary, these results derived from gain-of-function study of miR-155 suggest that miR-155 plays pivotal roles in regulating material metabolism in liver.

### 9. Overexpression of miR-155 in transgenic mouse liver ameliorated HFD-induced fatty liver

**Fig 6:**
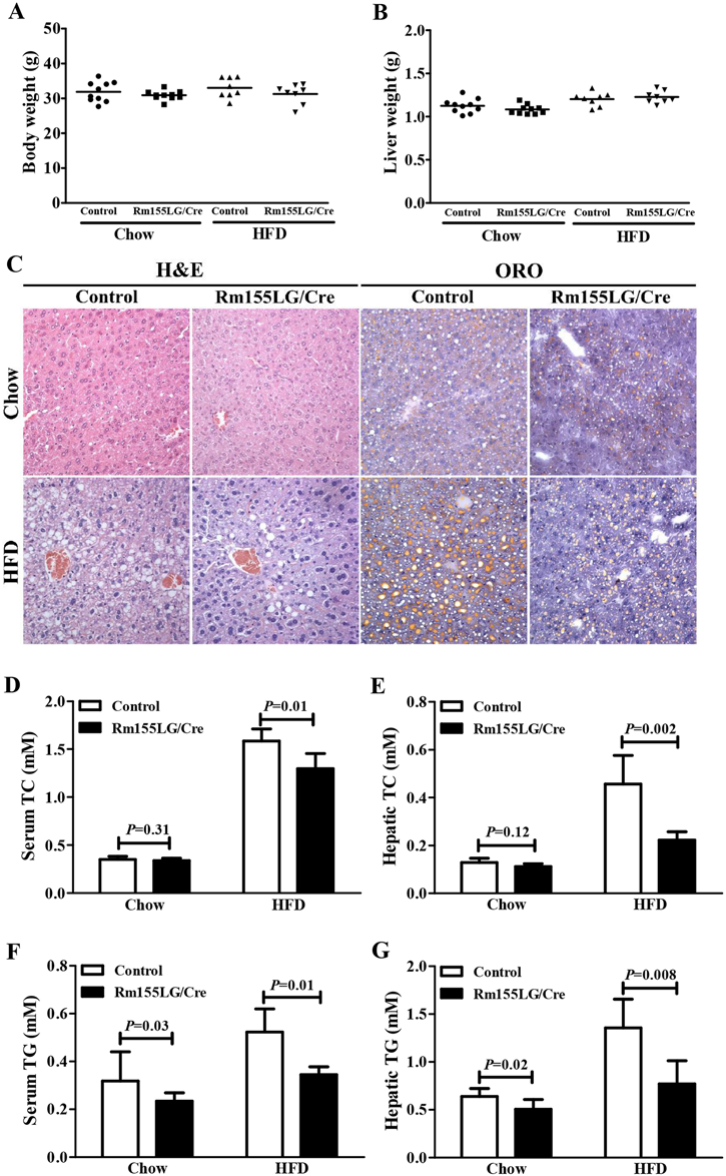
Enforced expression of miR-155 in the liver of Rm155LG/Alb-Cre mice improved HFD-induced hepatic steatosis. **(A)** Body weight of Rm155LG/Alb-Cre mice and controls fed normal chow diet or HFD. **(B)** Liver weight of Rm155LG/Alb-Cre mice vs. controls fed normal chow diet or HFD. **(C)** H&E staining and ORO staining of liver sections from control and Rm155LG/Alb-Cre mice. **(D-G)** Quantification of TC and TG in the serum and liver of control and Rm155LG/Alb-Cre mice fed either chow diet or HFD. Data are mean±SD (n = 6–8). Other details as in Fig. 3.

Since hepatic-specific overexpression of miR-155 in transgenic mice reduced hepatic and serum lipid profiles (Fig. 3, Fig. 4 and Fig. 5), we next tested whether miR-155 is connected to HFD-induced development of hepatic steatosis. To address this purpose, we fed male control and Rm155LG/Alb-Cre mice a HFD for 6 months. Under conditions of diet-induced obesity for 6 months, there was no evident difference in the final body weight and liver weight between Rm155LG/Alb-Cre mice and control mice fed HFD (Fig. 6A, B). We next examined histologic changes of the livers of Rm155LG/Alb-Cre mice and control mice fed chow or HFD. H&E staining of liver sections showed that livers of HFD-fed control mice had numerous diffused intracellular lipid droplets compared with Rm155LG/Alb-Cre mice fed HFD (Fig. 6C). Oil Red O staining of lipids further confirmed a massive accumulation of neutral lipids in the livers of HFD-fed control mice but not in the livers of HFD-fed Rm155LG/Alb-Cre mice (Fig. 6C). Biochemical analysis demonstrated that hepatic and serum TC and TG contents were significantly increased in control mice vs Rm155LG/Alb-Cre mice fed HFD (Fig. 6D-G). Taken together, these results indicate that liver-specific overexpression of miR-155 in transgenic mice improves HFD-induced steatotic phenotype in the liver.

### 10. Ces3/TGH, a regulator of lipid metabolism, is a direct target of miR-155 in liver

**Fig 7:**
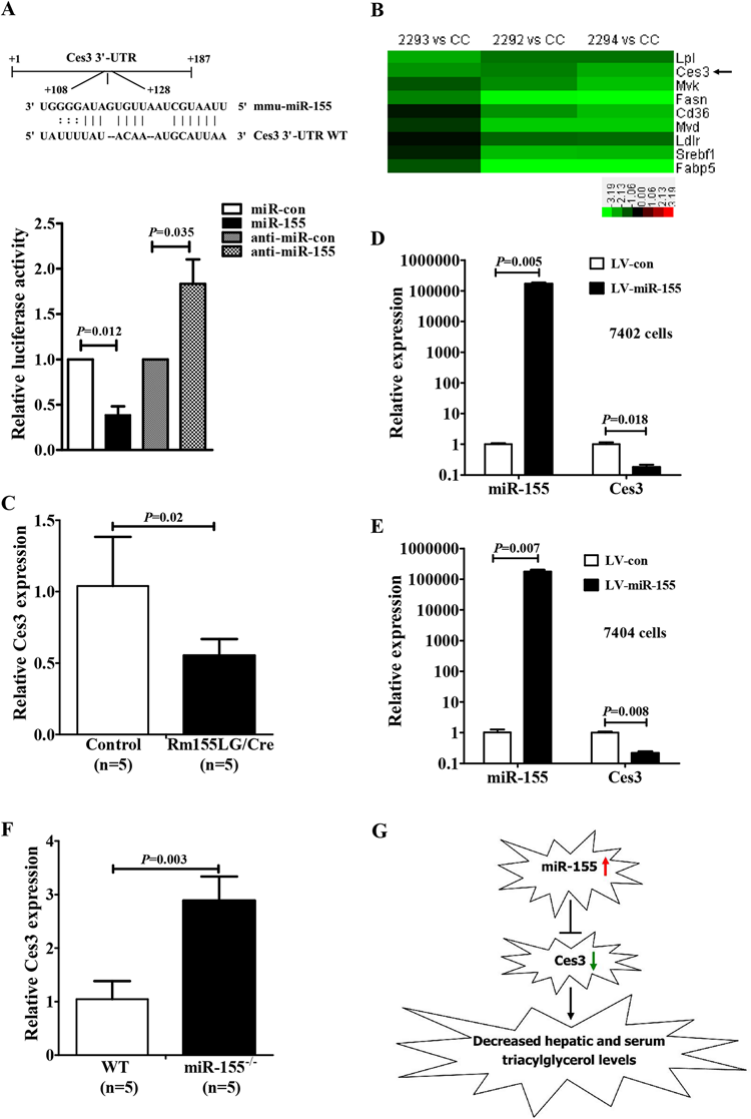
Identification of Ces3/TGH as a miR-155 target gene. **(A)** Ces3 is a target gene of miR-155. The luciferase reporter assay was performed using Hepa1–6 cells as described in the Materials and methods section. **(B)** Microarray revealed the reduced expression of Ces3 in the liver of Rm155LG/Alb-Cre mice. A cluster heat map for 9 hepatic lipid metabolism-related genes is shown. Other details as in S2 Fig. **(C)** qRT-PCR analysis of Ces3 expression in Rm155LG/Alb-Cre mouse liver. **(D-E)** qRT-PCR assay for Ces3 expression in vector- and miR-155-expressing 7402 (D) and 7404 (E) cells. **(F)** qRT-PCR analysis of Ces3 expression in miR-155 knockout mouse liver. **(G)** Schematic diagram indicating the pathway of miR-155-mediated downregulation of Ces3 expression leading to reduced hepatic and blood TG levels.

Next, we further explored the direct molecular mechanisms underlying such pleiotropic effects of miR-155 in liver. It is generally accepted that miRNAs exert their function by regulating expression of their downstream target gene(s). Thus, putative miR-155 targets involved in these above-mentioned functions of miR-155 were predicted by using common databases, such as microRNA.org, RNAhybrid and miRWalk. These databases predicted that carboxylesterase 3/triacylglycerol hydrolase (Ces3/TGH) is a potential target of miR-155. The 3’-UTR of Ces3 mRNA contains a complementary site for the seed region of miR-155 (Fig. 7A). Notably, previous studies have shown that an ER-localized TGH, also termed Ces3 in mice and carboxylesterase 1 in humans, is highly expressed in the liver and adipose tissue [20] and has been suggested to play an important role in lipid metabolism, especially TG metabolism [21–23].

We further performed luciferase reporter assay to determine whether miR-155 can directly target the 3’-UTR of Ces3. Transient transfection of wild-type Ces3-luc reporter with miR-155 mimics into NIH3T3 cells led to a significant decrease in luciferase activity compared with mimics control (Fig. 7A). Further, microarray analysis revealed the downregulation of Ces3 expression in the liver of Rm155LG/Alb-Cre mice (Fig. 7B), while qRT-PCR analysis demonstrated that Ces3 mRNA levels in the liver of Rm155LG/Alb-Cre mice were significantly down-regulated (Fig. 7C), and ectopic expression of miR-155 in BEL-7402 (Fig. 7D) and BEL-7404 (Fig. 7E) human hepatocellular carcinoma cell lines resulted in significant reduction of endogenous Ces3. On the other hand, Ces3 levels in the liver of miR-155^-/-^ mice were significantly up-regulated (Fig. 7F). Therefore, miR-155 negatively regulates Ces3 expression in mouse liver. More importantly, these aforementioned results suggest that Ces3 is a direct target of miR-155.

The previous studies have shown that Ces3/TGH knockout mice presented with reduced hepatic and serum TG contents [23], while serum TG level was increased in transgenic mice that express human TGH specifically in the liver [24], clearly indicating that Rm155LG/Alb-Cre mice (this study) and Ces3/TGH knockout mice [23] exhibited the same change tendency in hepatic and serum TG levels. Altogether all these results suggest that Ces3/TGH is a direct miR-155 target gene that is likely at least partially responsible for serum and hepatic TG lowering observed in Rm155LG/Alb-Cre mice (Fig. 7G).

## Discussion

In 2006, the lab of Prof. Carlo M. Croce generated Eμ-miR155 transgenic mice in which the expression of mmu-miR-155 (mouse miR-155) is under the control of a VH promoter-Ig heavy chain Eμ enhancer, which becomes active at the late pro-B cell stage of the B cell development [12], while in 2013, transgenic mice (i.e., miR155TG mice) overexpressing miR-155 under control of the ubiquitous phosphoglycerate kinase (PGK) promoter were generated [8]. In this study, we generated a novel conditional Rm155LG transgenic mouse line expressing a mouse miR-155 transgene using Cre/lox P system. Compared with above-mentioned Eμ-miR155 transgenic mice [12] and miR155TG transgenic mice [8], Rm155LG transgenic mice generated in this study demonstrate many advantages and wide applications described below.

Firstly, the conditional Rm155LG transgenic mice have the potential to be employed in a wide range of studies by crossing with various cell/tissue-specific Cre mouse lines (http://nagy.mshri.on.ca/cre_new/index.php) to realize the cell/tissue/organ-specific overexpression of miR-155 and Luc transgenes. In this study, we demonstrated the liver-specific over-expression of the miR-155 and Luc transgenes in Rm155LG/Alb-Cre mice using Alb-Cre mice. Secondly, as both Luc and miR-155 transgenes can be “switched-on” simultaneously in a Cre-dependent manner in the same cells, tissues or organs of Rm155LG transgenic mice, Rm155LG transgenic mice enable bioluminescence imaging to noninvasively monitor tumor initiation, growth, progression, regression, relapse and therapeutic response in vivo with a noninvasive in vivo bioluminescence imaging approach, as strongly supported by other findings [25–31]. There are several lines of evidence that miR-155 was found to be overexpressed in lymphoma, leukemia and several solid tumors [i.e., nasopharyngeal carcinoma (NPC), breast cancer, lung cancer, colon cancer, cervical cancer, pancreatic ductal adenocarcinoma (PDAC), thyroid carcinoma, and head and neck squamous cell carcinomas] [3,32,33], indicating that it might play a significant role in the process of carcinogenesis, acting predominantly as an oncomiR. A direct evidence that miR-155 is involved in B-cell lymphoma had been obtained experimentally in Eμ-miR155 transgenic mice harboring mouse miR-155 transgene (under the control of a VH promoter) that is specifically over-expressed in B-cell lineage [12], whereas until now, the a direct evidence that miR-155 as oncomiR can also cause the mentioned-above solid tumors is not obtained experimentally in transgenic mice. Therefore, the combinational uses of Rm155LG transgenic mice and various cell/tissue-specific Cre mouse lines will be able to provide us with a powerful approach to uncover whether miR-155 as oncomiR can also initiate the mentioned-above solid tumors in transgenic mice. Finally, as mentioned above, miR-155 is a typical multifunctional miRNA involved in various physiological and pathological processes such as haematopoietic lineage differentiation, immunity, inflammation, cancer, and cardiovascular diseases [3], Rm155LG transgenic mice will be combined with miR-155 knockout mice [34] to further insight into the roles of miR-155 in the aforementioned processes.

**Fig 8:**
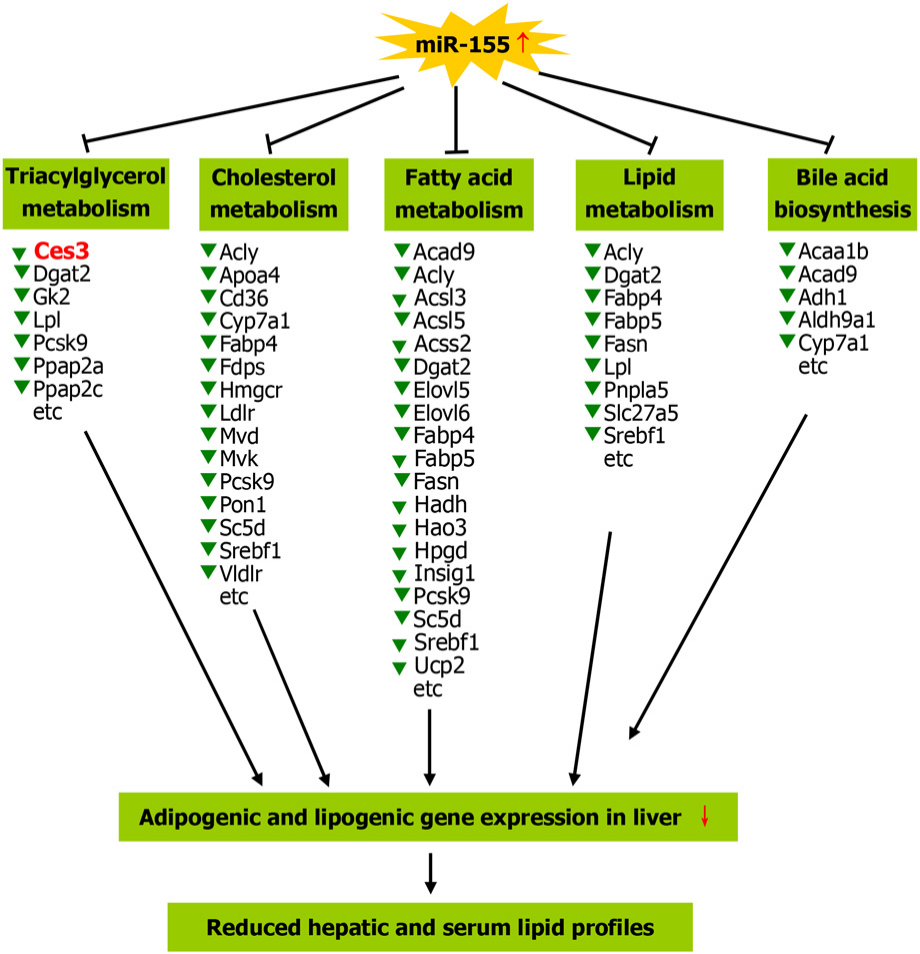
A proposed model on miR-155 overexpression reducing hepatic and blood lipid profiles. The enforced expression of miR-155 in the liver of transgenic mice is able to induce a general downward trend in the expression profile of hepatic genes involved lipogenesis, fatty acid metabolism, triacylglycerol metabolism, cholesterol metabolismn and bile acid biosynthesis, etc, thereby causing the decreased hepatic lipid content by decreasing adipogenic and lipogenic gene expression in liver, which reduces blood lipid concentration. Other details as in S6 Table and S7 Table.

As described in Introduction section, miR-155 is involved in adipocyte differentiation, adipogenesis and obese [5–8], suggesting that miR-155 might be involved in lipid metabolism. It is well known that the liver is an important place for a variety of material metabolism, as well as an important place for lipid metabolism (e.g., fatty acid, triglyceride and cholesterol metabolism). The liver plays important roles in the process of metabolism of lipid digestion, absorption, decomposition, synthesis, storage and transportation, while lipid metabolism is modulated by various hepatic metabolism-related enzymes. In this study, we explored the influences of liver-specific overexpression of miR-155 transgene in Rm155LG/Alb-Cre transgenic mice on lipid metabolism. Our data derived from miR-155 gain of function study demonstrated that Rm155LG/Alb-Cre mice exhibited decreased levels of serum TC, TG and HDL, and hepatic lipid, TG, HDL and FFA, which was accompanied by a general downward trend in the expression of hepatic genes (e.g., Acly, Fasn, Elovl5, Elovl6, Ucp2, Acss2, Acsl5, Ces3, Fabp4, Mvk, Mvd, Insig1, Ppap2a, Dgat2, Ppap2c, Pcsk9, Lpl, Gk2, Apoa4, Cd36 and Ldlr) involved in lipogenesis, lipid transport, lipid storage, bile acid biosynthesis, fatty acid synthesis, fatty acid oxidation, fatty acid catabolism, cholesterol biosynthesis, cholesterol transport, cholesterol homeostasis and triglyceride synthesis, indicating that the decreased expression of hepatic genes involved in lipid metabolism might be responsible, or contribute to the altered hepatic and serum lipid profiles (Fig. 8).

miRNAs are critical modulators of many pathways by negatively regulating the expression of multiple target genes. Our studies suggested that Ces3 is a direct target negatively regulated by miR-155 in mouse liver. As mentioned above, Ces3 plays an important role in lipid metabolism, especially TG metabolism [21–23]. Several previous studies have demonstrated that Ces3 knockout mice showed reduced hepatic and serum TG contents [23], while conversely, serum TG level was increased in transgenic mice with liver-specific expression of human TGH transgene [24]. In this study, we found that hepatic-specific overexpression of miR-155 transgene in mice led to the reduced hepatic and serum TG concentrations, accompanied by the deceased expression of Ces3 in Rm155LG/Alb-Cre mouse liver. Thus, although there may be more miR-155 targets, such as NR1H3 (liver X receptor α, LXRa) [10], that likely contribute to lipid metabolism, herein we show, for what we believe is the first time, that repression of Ces3 expression in the liver of Rm155LG/Alb-Cre mice does represent a plausible mechanism by which the hepatic and blood phenotypes (i.e., TG lowering) are induced (Fig. 7G).

Furthermore, LXRa was identified as a direct target gene of miR-155, and LXRa upregulation in the absence of repression by miR-155 in the liver of miR-155^-/-^ mice fed HFD resulted in upregulation of LXR-responsive genes (i.e., Fasn, Cd36, Lpl) and excessive TG and TC accumulation in liver, thereby contributing to fatty liver of miR-155^-/-^ mice fed HFD [10]. Therefore, a loss of repression of LXRa expression in miR-155^-/-^ mice does represent a plausible mechanism by which the above-mentioned hepatic phenotypes are induced [10]. In addition, we also observed that the LXR-responsive genes (i.e., Fasn, Cd36, Lpl, Cyp7a1 and Srebf1) (Fig. 4A, Fig. 5, Fig. 7B, S6 Table and S7 Table) were downregulated in the liver of Rm155LG/Alb-Cre mice. In lipase (Lpl)-knockout mice, plasma free fatty acid and TG levels were low, associated with low liver TG content [35]. Previous studies indicated that hepatic CD36/fatty acid translocase expression were abnormally increased in non-alcoholic fatty liver disease (NAFLD), the forced expression of hepatic CD36 increased hepatic triglyceride storage and plasma triglyceride levels in mice fed a standard chow diet, and increased expression and function of CD36 in hepatocytes could contribute to steatosis not only in rodents but also in humans [36–38]. Thus, we speculate that the reduced expression of LXR-responsive genes (i.e., Cd36 and Lpl) induced by the enforced miR-155 expression is likely at least partially responsible for hepatic and serum lipid lowering observed in Rm155LG/Alb-Cre mice.

Moreover, the liver-specific miR-155 overexpression in Rm155LG/Alb-Cre mice down-regulated cholesterol transporter protein Apoa4 in liver and significantly reduced serum TC and HDL levels. Apoa4^-/-^ mice exhibited decreased serum HDL levels[39], while Apoa4 transgenic mice showed increased serum HDL levels [40]. Thus, the reduced Apoa4 expression is likely at least partially responsible for serum HDL lowering observed in Rm155LG/Alb-Cre mice.

Miller et al reported that abundant lipid droplets were observed in livers of miR-155^-/-^ mice fed chow or HFD for 24 weeks compared to WT fed chow or HFD, respectively [10]. Furthermore, the levels of serum VLDL/LDL (very low-density lipoprotein/low-density lipoprotein) cholesterol, and liver TG and TC were significantly higher in HFD-fed miR-155^-/-^ mice vs WT, accompanied by increased expression of hepatic genes involved in fatty acid uptake (Cd36) and lipid metabolism (Fasn, Fabp4, Lpl, Abcd2 and Pla2g7) [10]. These aforementioned results clearly indicated that Rm155LG/Alb-Cre mice (this study) and miR-155^-/-^ mice [10] exhibited the opposite change tendency in hepatic lipid metabolism gene expression, liver lipid content, and hepatic TG and TC levels. Surprisingly, a detailed analysis of hepatic and serum lipid compositions revealed that TG, TC and HDL levels in serum did not significantly differ between WT and miR-155^-/-^ mice fed chow or HFD for 24 weeks, and hepatic TG and TC did not significantly differ between WT and miR-155^-/-^ mice fed chow for 24 weeks [10], but in this study, Rm155LG/Alb-Cre mice fed chow was associated with significantly decreased serum TG, TC and HDL levels, and hepatic TG and HDL levels, suggesting that the opposite change tendency in levels of some hepatic and serum lipid compositions was not observed in miR-155^-/-^ mice, which awaits further investigation. Additionally, inhibition of miR-155 expression significantly induced lipid uptake, whereas miR-155 overexpression decreased lipid uptake in PMA-differentiated THP-1 cells and dendritic cells [11]. Summarily, gain-of-function and loss-of-function studies of us and other investigators fully demonstrate that miR-155 plays an important role in regulating lipid metabolism, and negatively modulates levels of hepatic and serum lipid compositions.

Mice lacking endogenous miR-155 that were fed HFD for 6 months developed increased hepatic steatosis compared to WT controls, accompanied by the significant increase in liver lipid droplets, hepatic TG and TC levels, and serum VLDL/LDL cholesterol levels [10], while in the present study, liver-specific overexpression of miR-155 transgene in Rm155LG/Alb-Cre mice led to decreased hepatic and serum lipid levels, and alleviated HFD-induced fatty liver. Moreover, several genes with increased mRNA levels in the liver of Nas1^-/-^ mice with fatty liver have a functional association with fatty acid metabolism (Gpam, Acsl5, Acly, Scd1, Elov16 and Apoa4) [41], while in Rm155LG/Alb-Cre mice, the decreased levels of hepatic lipid, TG, HDL and FAA were associated with a reduction in mRNA levels of hepatic genes Acsl5, Acly, Elov16 and Apoa4. These results suggest a protective role of miR-155 in the development of non-alcoholic hepatosteatosis in mice. Further experiments in primates will be required to evaluate the roles of miR-155 in improving hepatosteatosis or anti-hepatosteatosis in a HFD-fed animal model by using miR-155 mimics, which will be helpful studies in evaluating the prospects for therapeutical miR-155 gain of function to improve fatty liver in human.

In addition, hepatic miR-155 expression was increased in murine NAFLD [9], murine models of diet-induced obesity [10,42] and *ob/ob* mice on normal chow versus their respective controls [10], which appears to contradict a protective role of miR-155 in the development of non-alcoholic hepatosteatosis described above. Further study showed that in WT mice fed HFD for 24 weeks, miR-155 expression was higher in CD11b^+^ macrophages compared to the CD11b^-^ fraction, comprising of all other hepatic cell lineages [10]. These data suggest that homeostatic effects of miR-155 in liver are likely mediated by macrophages/Kupffer cells, and not by hepatocytes [10]. Miller et al pointed out that increased hepatic expression of miR-155 in models of NAFLD likely plays a critical homeostatic role designed to prevent excessive lipid accumulation in livers that can ultimately lead to liver damage [10], which warrants further investigation.

In this study, Rm155LG/Alb-Cre mice exhibited the significantly decreased levels of blood TG, TC and HDL, suggesting that gain of miR-155 function can produce a beneficial effect on serum TG, TC and HDL-cholesterol. In mice, unlike in humans, HDL makes up the majority of serum TC. Thus, further experiments in primates will be required to evaluate the relative effects of miR-155 gain of function on TG, TC, HDL and LDL in species that have lipoprotein profiles more similar to humans. These will be helpful studies in evaluating the prospects for therapeutical miR-155 overexpression by using miR-155 mimics to lower TG, TC and HDL in humans in addition to improving hepatosteatosis.

The altered lipid metabolism observed in both Rm155LG/Alb-Cre mice (this study) and miR-155^-/-^ mice [10] encouraged investigators to dissect the effects of gain or loss of miR-155 function on body weight and liver weight of these mice. In this study, Rm155LG/Alb-Cre mice fed chow showed the unaltered body weight and liver weight at indicated ages. Compared with WT controls, complete deficiency of miR-155 did not alter the final body weight of mice fed normal chow or HFD for 24 weeks, and the final liver weight of mice fed chow at 24 weeks, but mean liver weight was increased by 30% in miR-155^-/-^ mice fed HFD [10]. Additionally, the weight of epididymal fat pads did not differ between WT and miR-155^-/-^ mice fed chow or HFD, indicating a selective effect on liver [10]. These findings suggest that although miR-155 is involved in the regulation of lipid metabolism, miR-155 has little effect on body weight of mice fed chow or HFD.

## Conclusions

In summary, Rm155LG transgenic mice for Cre-mediated miR-155 conditional overexpression will constitute a very powerful tool to further dissect the multiple functions of miR-155 in vivo, which will facilitate studies on the roles of miR-155 in developmental, physiological, and pathological processes. Furthermore, liver-specific overexpression of miR-155 transgene in Rm155LG/Alb-Cre mice resulted in significantly reduced levels of serum TG, TC and HDL, as well as remarkably decreased contents of hepatic lipid, TG, HDL and FFA, which was accompanied by a general downward trend in the expression profile of hepatic genes involved lipid metabolism (e.g., lipogenesis, fatty acid synthesis, cholesterol biosynthesis and triglyceride synthesis) (Fig. 8). These data from miR-155 gain of function study demonstrate new model and insights to investigate the role of miR-155 in metabolic state of the liver. Although much more work is needed to understand all the roles of miR-155 in liver biology, lipid metabolism, hepatosteatosis, hepatic fibrosis, obesity and metabolic syndrome, and related molecular mechanisms, these results portend the mentioned-above therapeutic potential of miR-155 modulation.

## Supporting Information

S1 Fig mRFP expression in postnatal organs of Rm155LG transgenic mice. (DOC)

S2 Fig Class comparison and hierarchical clustering analysis of differentially expressed genes between Rm155LG/Alb-Cre and control mouse liver. (DOC)

S3 Fig Class comparison and hierarchical clustering analysis of differentially expressed genes involved in retinol metabolism and hepatic drug metabolism between Rm155LG/Alb-Cre and control mouse liver. (DOC)

S4 Fig Class comparison and hierarchical clustering analysis of differentially expressed genes involved in amino acid metabolism, nucleic acid metabolism and hormone metabolism between Rm155LG/Alb-Cre and control mouse liver. (DOC)

S1 Table Primers for qRT-PCR analysis of mouse miR-155. (DOC)

S2 Table List of primer pairs used for qRT-PCR analysis of lipid metabolism-related gene expression. (DOC)

S3 Table List of primer pairs used for qRT-PCR analysis of cholesterol and triacylglycerol metabolism-related gene expression. (DOC)

S4 Table Differentially expressed genes in liver between control and Rm155LG/Alb-Cre transgenic mice. (DOC)

S5 Table Differentially expressed genes involved in hepatic lipid, cholesterol and triacylglycerol metabolism between control and Rm155LG/Alb-Cre transgenic mice. (DOC)

S6 Table Hepatic lipid metabolism-related genes differentially expressed between control and Rm155LG/Alb-Cre transgenic mice (average of three biological replicates >2 fold-change, t-test p < 0.05). (DOC)

S7 Table Hepatic cholesterol and triacylglycerol metabolism-related genes differentially expressed between control and Rm155LG/Alb-Cre transgenic mice (average of three biological replicates >2 fold-change, t-test p < 0.05). (DOC)

S8 Table Gene ontology (GO) and KEGG pathway analysis of differentially expressed genes (related with hepatic lipid metabolism) from Rm155LG/Alb-Cre transgenic mice to control mice. (DOC)

S9 Table Vitamin, amino acid, nucleic acid, hormone metabolism- and hepatic drug metabolizing enzyme (DME)-related genes differentially expressed between control and Rm155LG/Alb-Cre transgenic mice (average of three biological replicates >2 fold-change, t-test p<0.05). (DOC)

S10 Table Gene ontology (GO) and KEGG pathway analysis of differentially expressed genes (related with metabolism of amino acids, nucleic acids, vitamins, drugs and hormones, etc) from Rm155LG/Alb-Cre transgenic mice to control mice. (DOC)
