# Supplementary material for: Overexpression of miR-155 in the Liver of Transgenic Mice Alters the Expression Profiling of Hepatic Genes Associated with Lipid Metabolism
Source: PLoS One. 2015 Mar 23;10(3):e0118417. doi: 10.1371/journal.pone.0118417 (PMC4370457; doi:10.1371/journal.pone.0118417)
Supplement: S1 Fig — (DOC) [file pone.0118417.s001.doc]

**Figures S1**

**A**


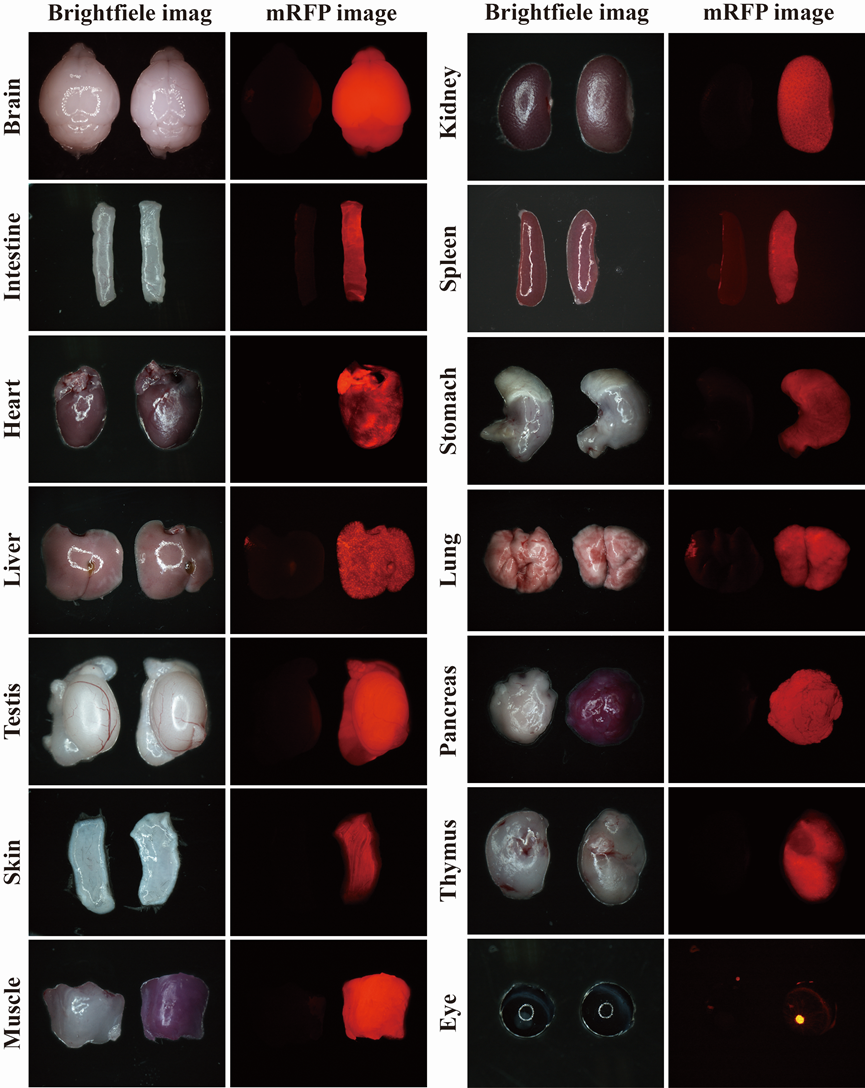


**
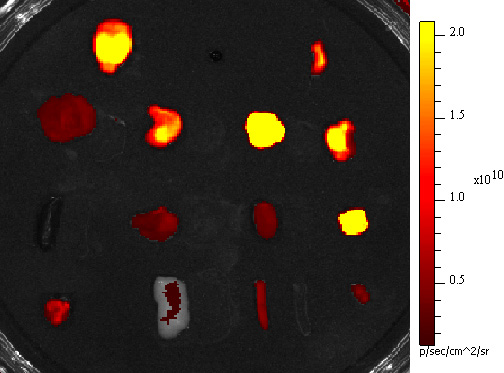

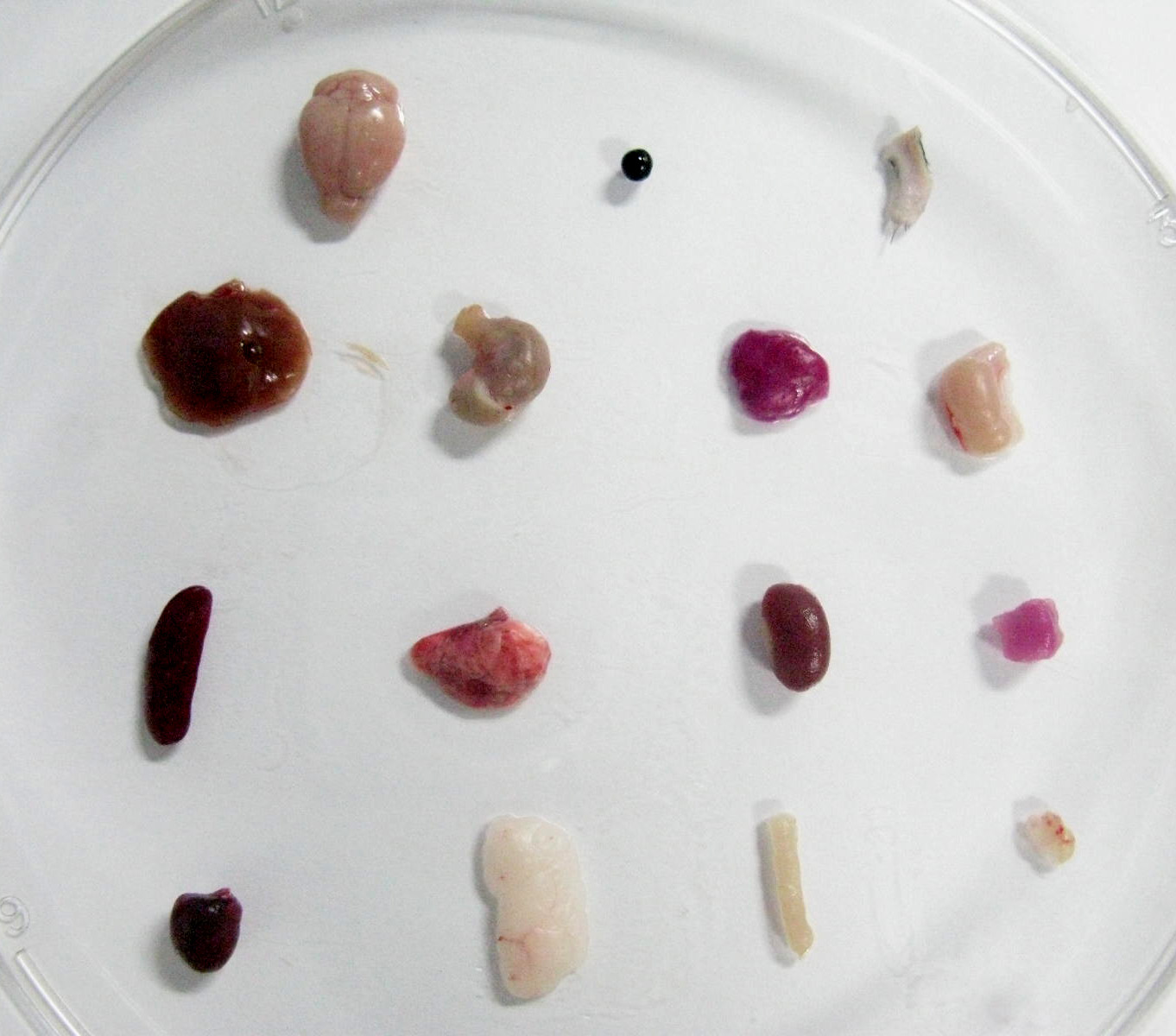
**

**B**

**C**

**Figure S1. mRFP expression in postnatal organs of Rm155LG transgenic mice**

**(A) Assay of mRFP expression in the postnatal organs of Rm155LG transgenic mice under stereo fluorescent microscope (Nikon, AZ100)**. The left organ samples in each figure were obtained from one non-transgenic littermate, while the right organ samples in each figure were obtained from one Rm155LG transgenic mouse. Muscle and pancreas from Rm155LG transgenic mice (the right samples in each figure) can be distinguished from their wildtype littermates according to their deep red color under daylight.

**(B-C) Different expression level of mRFP in postnatal organs of Rm155LG transgenic mice**The level of mRFP expression in the different postnatal organs of Rm155LG transgenic mice were detected by using the Xenogen IVIS Lumina Imaging System. B: photos taken with the Xenogen IVIS Lumina Imaging System, while C: the same samples (shown in Figure S1B) observed under brightfield.
