# Supplementary material for: Overexpression of miR-155 in the Liver of Transgenic Mice Alters the Expression Profiling of Hepatic Genes Associated with Lipid Metabolism
Source: PLoS One. 2015 Mar 23;10(3):e0118417. doi: 10.1371/journal.pone.0118417 (PMC4370457; doi:10.1371/journal.pone.0118417)
Supplement: S2 Fig — (DOC) [file pone.0118417.s002.doc]

**Figure S2**

**
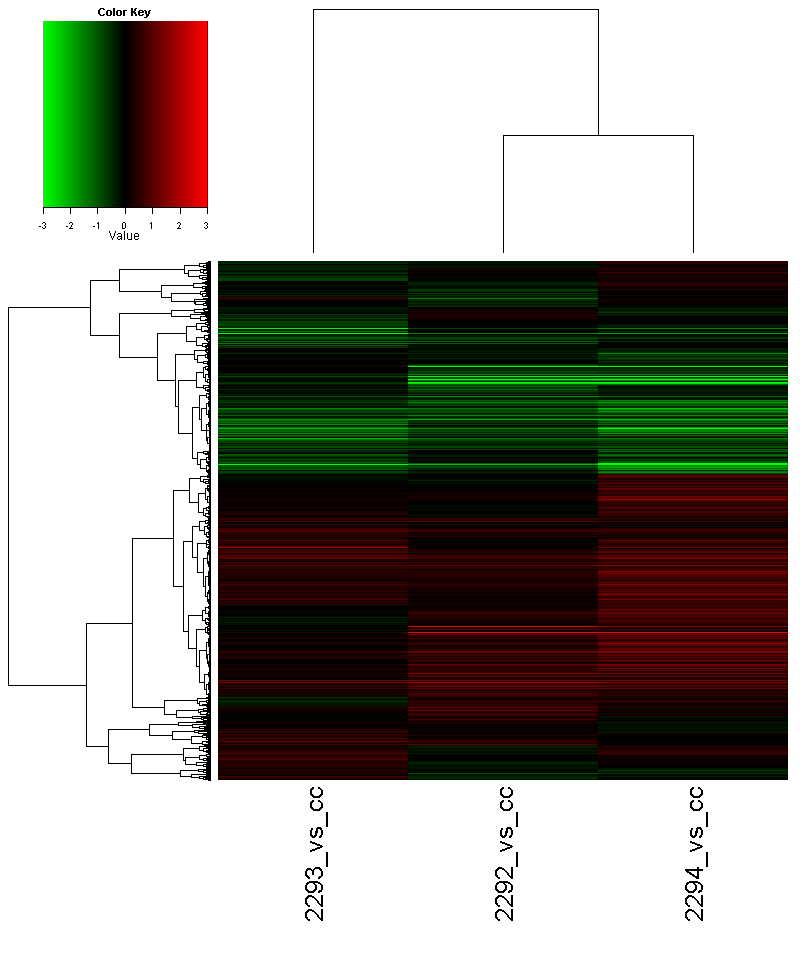
**

**Figure S2. Class comparison and hierarchical clustering analysis of differentially expressed genes between Rm155LG/Alb-Cre and control mouse liver.**

2292, 2293 and 2294 represented the total RNA (used in microarray experiment) isolated from the livers of three 4-month-old Rm155LG/Alb-Cre transgenic mice, while cc represented the pooled total RNA (used in microarray experiment) isolated from the livers of three control littermates. Equal amounts of total RNA from each control liver vole were pooled to prepare cc. 2292_vs_cc: 2292 compared to pooled cc; 2293_vs_cc: 2293 compared to pooled cc; 2294_vs_cc: 2294 compared to pooled cc. Only genes showing a fold change of more than 2 and a t test P value of less than 0.05 were included in the analysis. Red indicates increased expression; blue indicates reduced expression The mRNA microarray analysis showed that 638 mRNAs were differentially expressed between Rm155LG/Alb-Cre and control mouse liver.
