# Supplementary material for: Overexpression of miR-155 in the Liver of Transgenic Mice Alters the Expression Profiling of Hepatic Genes Associated with Lipid Metabolism
Source: PLoS One. 2015 Mar 23;10(3):e0118417. doi: 10.1371/journal.pone.0118417 (PMC4370457; doi:10.1371/journal.pone.0118417)
Supplement: S3 Fig — (DOC) [file pone.0118417.s003.doc]

**Figure S3**


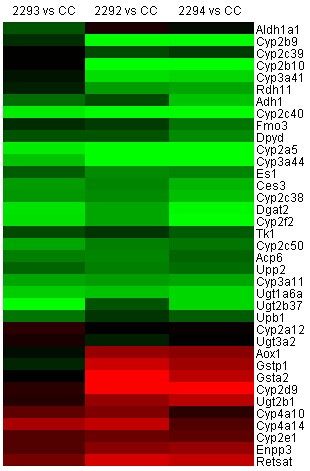

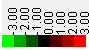


**Figure S3. Class comparison and hierarchical clustering analysis of differentially expressed genes involved in retinol metabolism and hepatic drug metabolism between Rm155LG/Alb-Cre and control mouse liver.**

A cluster heat map for differentially expressed genes (see Table S9 and Table S10) involved in retinol metabolism and hepatic drug metabolism is shown. Other details as in Figure S2.
