# Supplementary material for: Overexpression of miR-155 in the Liver of Transgenic Mice Alters the Expression Profiling of Hepatic Genes Associated with Lipid Metabolism
Source: PLoS One. 2015 Mar 23;10(3):e0118417. doi: 10.1371/journal.pone.0118417 (PMC4370457; doi:10.1371/journal.pone.0118417)
Supplement: S4 Fig — (DOC) [file pone.0118417.s004.doc]

**Figure S4**


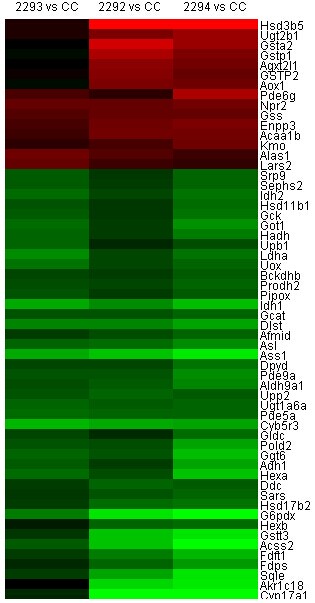

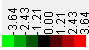


**Figure S4. Class comparison and hierarchical clustering analysis of differentially expressed genes involved in amino acid metabolism, nucleic acid metabolism and hormone metabolism between Rm155LG/Alb-Cre and control mouse liver.**

A cluster heat map for differentially expressed genes (see Table S9 and Table S10) involved in amino acid metabolism, nucleic acid metabolism and hormone metabolism is shown. Other details as in Figure S2.
