# Supplementary material for: Overexpression of miR-155 in the Liver of Transgenic Mice Alters the Expression Profiling of Hepatic Genes Associated with Lipid Metabolism
Source: PLoS One. 2015 Mar 23;10(3):e0118417. doi: 10.1371/journal.pone.0118417 (PMC4370457; doi:10.1371/journal.pone.0118417)
Supplement: S1 Table — (DOC) [file pone.0118417.s005.doc]

**Table S1. Primers for qRT-PCR analysis of mouse miR-155**

| **Primer name** | **Primer sequence** |
| --- | --- |
| U6 snRNA-RT | AACGCTTCACGAATTTGCGT |
| U6 snRNA-forward primer | CTCGCTTCGGCAGCACA |
| U6 snRNA-reverse primer | AACGCTTCACGAATTTGCGT |
| miR155-RT primer | GTCGTATCCAGTGCAGGGTCCGAGGTATTCGCACTGGATACGACACCCC |
| miR155-forward primer | GACTGTTAATGCTAATCGTGATAG |
| miR155-reverse primer  mGAPDH- forward primer  mGAPDH- reverse primer | GTGCAGGGTCCGAGGTATTC  AGGTCGGTGTGAACGGATTTG  GGGGTCGTTGATGGCAACA |
