# Supplementary material for: Overexpression of miR-155 in the Liver of Transgenic Mice Alters the Expression Profiling of Hepatic Genes Associated with Lipid Metabolism
Source: PLoS One. 2015 Mar 23;10(3):e0118417. doi: 10.1371/journal.pone.0118417 (PMC4370457; doi:10.1371/journal.pone.0118417)
Supplement: S2 Table — (DOC) [file pone.0118417.s006.doc]

**Table S2. List of primer pairs used for qRT-PCR analysis**

**of lipid metabolism-related gene expression**

| **Gene** | **Forward Primer (5’-3’)** | **Reverse Primer (5’-3’)** |
| --- | --- | --- |
| Acaa1a | TCTCCAGGACGTGAGGCTAAA | CGCTCAGAAATTGGGCGATG |
| Acaa1b | CAGGACGTGAAGCTAAAGCCT | CTCCGAAGTTATCCCCATAGGAA |
| Acad9 | TCCAGAGGTCAGTCAACATGA | CCTGGTCAATTTTTCGAGAGTCC |
| Acat2 | CCCGTGGTCATCGTCTCAG | GGACAGGGCACCATTGAAGG |
| Acot1 | ATACCCCCTGTGACTATCCTGA | CAAACACTCACTACCCAACTGT |
| Acsl3 | AACCACGTATCTTCAACACCATC | AGTCCGGTTTGGAACTGACAG |
| Acsl5 | TCCTGACGTTTGGAACGGC | CTCCCTCAATCCCCACAGAC |
| Adh1 | GCAAAGCTGCGGTGCTATG | TCACACAAGTCACCCCTTCTC |
| Adipor1 | TCTTCGGGATGTTCTTCCTGG | TTTGGAAAAAGTCCGAGAGACC |
| Adipor2 | GGAGTGTTCGTGGGCTTAGG | GCAGCTCCGGTGATATAGAGG |
| Asah1 | CGTGGACAGAAGATTGCAGAA | TGGTGCCTTTTGAGCCAATAAT |
| Cidec | ATGGACTACGCCATGAAGTCT | CGGTGCTAACACGACAGGG |
| Cpt1c | TCTTCACTGAGTTCCGATGGG | ACGCCAGAGATGCCTTTTCC |
| Cxcl16 | CCTTGTCTCTTGCGTTCTTCC | TCCAAAGTACCCTGCGGTATC |
| Cyp4a10 | TTCCCTGATGGACGCTCTTTA | GCAAACCTGGAAGGGTCAAAC |
| Cyp4a12a | CCTCTAATGGCTGCAAGGCTA | CCAGGTGATAGAAGTCCCATCT |
| Cyp4a12b | GGGGAGATCAGACCCAAAAGC | ATTCGTCGGTGCTGAAACCAT |
| Cyp4a14 | TTTAGCCCTACAAGGTACTTGGA | GCAGCCACTGCCTTCGTAA |
| Cyp8b1 | CCTCTGGACAAGGGTTTTGTG | GCACCGTGAAGACATCCCC |
| Ces3 | AAGCTCCTAGCAAACAAGCAA | TGGGCTAATAAGGGCCTTGAA |
| Ces3(h) | TCCAGCATCGACCCAGTTCT | GGACCTCCGAACACAAAAGCA |
| Degs1 | GAATGGGTCTACACGGACCAG | CGAGAAGCATCATGGCTACAA |
| Dgat2 | GCGCTACTTCCGAGACTACTT | GGGCCTTATGCCAGGAAACT |
| Elovl5 | ATGGAACATTTCGATGCGTCA | GTCCCAGCCATACAATGAGTAAG |
| Elovl6 | GAAAAGCAGTTCAACGAGAACG | AGATGCCGACCACCAAAGATA |
| Fabp1 | ATGAACTTCTCCGGCAAGTACC | CTGACACCCCCTTGATGTCC |
| Fabp2 | GTGGAAAGTAGACCGGAACGA | CCATCCTGTGTGATTGTCAGTT |
| Fabp3 | ACCTGGAAGCTAGTGGACAG | TGATGGTAGTAGGCTTGGTCAT |
| Fabp6 | CTTCCAGGAGACGTGATTGAAA | CCTCCGAAGTCTGGTGATAGTTG |
| Fads2 | AAGGGAGGTAACCAGGGAGAG | CCGCTGGGACCATTTGGTAA |
| Fasn | GGAGGTGGTGATAGCCGGTAT | TGGGTAATCCATAGAGCCCAG |
| Fdft1 | ATGGAGTTCGTCAAGTGTCTAGG | CGTGCCGTATGTCCCCATC |
| Gba | GCCAGGCTCATCGGATTCTTC | CACGGGGTCAAGAGAGTCAC |
| Gm2a | CGCCTTTCCCAACTTGGTG | TGACGACTACATCTCCAGGAAC |
| Hacl1 | AAGTCATCGCCCAGGCTCTAA | GAGACCGGGACCAGAAACAAC |
| Hadhb | ACTACATCAAAATGGGCTCTCAG | AGCAGAAATGGAATGCGGACC |
| Hao2 | GCAGACTTTAAGGCACAAGCA | TGCCAAGTTGTCATTGTAGGTT |
| Hpgd | GTGAACGGCAAAGTGGCTCT | TCCAATCCACCAATGCTACCT |
| Hsd11b1 | CAGAAATGCTCCAGGGAAAGAA | GCAGTCAATACCACATGGGC |
| Lipg | ATGCGAAACACGGTTTTCCTG | GTAGCTGGTACTCCAGTGGG |
| Lrpap1 | CACAACCTCAACGTCATCCTG | AGCACATTGTACTCCTGGATCTT |
| Me1 | GTCGTGCATCTCTCACAGAAG | TGAGGGCAGTTGGTTTTATCTTT |
| Mttp | CTCTTGGCAGTGCTTTTTCTCT | GAGCTTGTATAGCCGCTCATT |
| Pcsk9 | GAGACCCAGAGGCTACAGATT | AATGTACTCCACATGGGGCAA |
| Pex7 | CCGAGTTCTCTCCGTACCTG | ACGTCAAACAAGCCGTCATTC |
| Plcl2 | GGGCTCTGAACTCAAAAAGGT | AAATGCCATTGCTGCGGAATA |
| Sc4mol | AAACAAAAGTGTTGGCGTGTTC | AAGCATTCTTAAAGGGCTCCTG |
| Sc5d | TCTCAGTGCCGCCGATTACTA | CTTGACAGTGAACACGATCTCA |
| Scarf1 | GGAGTGCAGTCGTAAGTGCC | CAGCTCACAGCGGATTCCAT |
| Slc27a2 | TCCTCCAAGATGTGCGGTACT | TAGGTGAGCGTCTCGTCTCG |
| Slc27a3 | GGCCCGGATTTCCTTTGGATT | CCCATAGGTGGAGCCCCAT |
| Slc27a4 | ACTGTTCTCCAAGCTAGTGCT | GATGAAGACCCGGATGAAACG |
| Slc27a5 | CTACGCTGGCTGCATATAGATG | CCACAAAGGTCTCTGGAGGAT |
| Slc27a6 | CTCCAACCTTCGCTTCGATTC | TCTGACGTGTTTTGGGAGACT |
| Slco1a1 | GTGCATACCTAGCCAAATCACT | CCAGGCCCATAACCACACATC |
| Slco1a4 | GCTTTTCCAAGATCAAGGCATTT | CGTGGGGATACCGAATTGTCT |
| SREBF1 | GCAGCCACCATCTAGCCTG | CAGCAGTGAGTCTGCCTTGAT |
| Sult2a2 | TAACTTACCCCAAGTCAGGAACG | ATGGGAAGATGGGAGGTTATGA |
| Vldlr | GGCAGCAGGCAATGCAATG | GGGCTCGTCACTCCAGTCT |
