# Supplementary material for: Overexpression of miR-155 in the Liver of Transgenic Mice Alters the Expression Profiling of Hepatic Genes Associated with Lipid Metabolism
Source: PLoS One. 2015 Mar 23;10(3):e0118417. doi: 10.1371/journal.pone.0118417 (PMC4370457; doi:10.1371/journal.pone.0118417)
Supplement: S3 Table — (DOC) [file pone.0118417.s007.doc]

**Table S3. List of primer pairs used for qRT-PCR analysis**

**of cholesterol and triacylglycerol metabolism-related gene expression**

| **Gene** | **Forward Primer (5’-3’)** | **Reverse Primer (5’-3’)** |
| --- | --- | --- |
| Abca1 | GCTTGTTGGCCTCAGTTAAGG | GTAGCTCAGGCGTACAGAGAT |
| Abcg1 | CTTTCCTACTCTGTACCCGAGG | CGGGGCATTCCATTGATAAGG |
| Angptl3 | GAGGAGCAGCTAACCAACTTAAT | TCTGCATGTGCTGTTGACTTAAT |
| Apoa1 | GGCACGTATGGCAGCAAGAT | CCAAGGAGGAGGATTCAAACTG |
| Apoa2 | TGGTCGCACTGCTGGTAAC | TTTGCCATATTCAGTCATGCTCT |
| Apoa4 | CCAATGTGGTGTGGGATTACTT | AGTGACATCCGTCTTCTGAAAC |
| Apob | TTGGCAAACTGCATAGCATCC | TCAAATTGGGACTCTCCTTTAGC |
| Apoe | CTGACAGGATGCCTAGCCG | CGCAGGTAATCCCAGAAGC |
| Cyb5r3 | CAGGGCTTCGTGAATGAGGAG | TCCACACATCAGTATCAGCGG |
| Cyp39a1 | TGCACTGCATGAGCGACTG | GGTATTGAGTGTGGCTGGATAAA |
| Cyp46a1 | AGCCGCTATGAGCACATCC | CCATACTTCTTAGCCCAATCCAG |
| Cyp51 | GACAGGAGGCAACTTGCTTTC | GTGGACTTTTCGCTCCAGC |
| Cyp7b1 | GGAGCCACGACCCTAGATG | TGCCAAGATAAGGAAGCCAAC |
| Fdft1 | ATGGAGTTCGTCAAGTGTCTAGG | CGTGCCGTATGTCCCCATC |
| Fdps | GGAGGTCCTAGAGTACAATGCC | AAGCCTGGAGCAGTTCTACAC |
| Gk2 | GCCTCGAAGCAAACCTCTG | TGTGTCAGTTCCACCTGATGA |
| Gpd1 | ATGGCTGGCAAGAAAGTCTG | CGTGCTGAGTGTTGATGATCT |
| Gpd2 | GAAGGGGACTATTCTTGTGGGT | GGATGTCAAATTCGGGTGTGT |
| Gyk | TGAACCTGAGGATTTGTCAGC | CCATGTGGAGTAACGGATTTCG |
| Hmgcs1 | AACTGGTGCAGAAATCTCTAGC | GGTTGAATAGCTCAGAACTAGCC |
| Hmgcs2 | GAAGAGAGCGATGCAGGAAAC | GTCCACATATTGGGCTGGAAA |
| Insig1 | CACGACCACGTCTGGAACTAT | TGAGAAGAGCACTAGGCTCCG |
| Insig2 | GGAGTCACCTCGGCCTAAAAA | CAAGTTCAACACTAATGCCAGGA |
| Lcat | GTAACCACACACGGCCTGTC | TCTTACGGTAGCACATCCAGTT |
| Ldlrap1 | CCTCAAGTACCTTGGTATGACGC | GAGGCTGTCGGTCAGGATG |
| Mvd | ATGGCCTCAGAAAAGCCTCAG | TGGTCGTTTTTAGCTGGTCCT |
| Mvk | GGTGTGGTCGGAACTTCCC | CCTTGAGCGGGTTGGAGAC |
| Npc1l1 | TGTCCCCGCCTATACAATGG | CCTTGGTGATAGACAGGCTACTG |
| Nsdhl | TCATGGTGAATCAAAGCGAGG | CCGGGGGTTATCAAAGCCTTG |
| Osbpl5 | TTCTGGGCTGCGAAAATGAG | GTCAGATCCATTGCATAGCCTG |
| Pcsk9 | GAGACCCAGAGGCTACAGATT | AATGTACTCCACATGGGGCAA |
| Pmvk | AAAATCCGGGAAGGACTTCGT | AGAGCACAGATGTTACCTCCA |
| Pon1 | GGTGTTGGCACTTTACAAGAACC | GGCGTTACTTCACGGAAAGC |
| Prkaa1 | GTCAAAGCCGACCCAATGATA | CGTACACGCAAATAATAGGGGTT |
| Prkaa2 | CAGGCCATAAAGTGGCAGTTA | AAAAGTCTGTCGGAGTGCTGA |
| Prkag2 | AAAGAACCCTAGCCTGAAGAGG | ACCTTCCGAGATGAATGCTTTT |
| Ppap2a | AGAGGGGCTTTTTCTGTACTGA | TATACGGGACGGGATGGTACT |
| Ppap2b | TCGTCCCTGAGAGTAAGAACG | TGCTTGTCTCGATGATGAGGAA |
| Ppap2c | CTCACGGTCCGCTATGTTTCA | GGTCAGCGTCAGTGACAGAC |
| Sc4mol | AAACAAAAGTGTTGGCGTGTTC | AAGCATTCTTAAAGGGCTCCTG |
| Scap | TGGAGCTTTTGAGACTCAGGA | TCGATTAAGCAGGTGAGGTCG |
| Scarf1 | TGGGACTAGAGCTGGTGTTCT | CAGATGGGGATGGTGCATTCT |
| Sqle | ATAAGAAATGCGGGGATGTCAC | ATATCCGAGAAGGCAGCGAAC |
| Stard3 | GTGACTTGGAGCGCAGTTTG | GCCAGTGTTGGTATTTAGCTCG |
| Tm7sf2 | AGCTTCGGGGGAGTCCTTC | CCAGGCACGGCCATACTTTT |
| Trerf1 | GAGAACCTCTTCTATCAACAGCC | AGCCCCAGAGATTGTATTCCC |
| Vldlr | GGCAGCAGGCAATGCAATG | GGGCTCGTCACTCCAGTCT |
