# Supplementary material for: Overexpression of miR-155 in the Liver of Transgenic Mice Alters the Expression Profiling of Hepatic Genes Associated with Lipid Metabolism
Source: PLoS One. 2015 Mar 23;10(3):e0118417. doi: 10.1371/journal.pone.0118417 (PMC4370457; doi:10.1371/journal.pone.0118417)
Supplement: S4 Table — (DOC) [file pone.0118417.s008.doc]

**Table S4. Differentially expressed genes in liver**

**between control and Rm155LG/Alb-Cre** transgenic mice

| **Differential expression** | **Number of genes** | **Fold difference (miR-155 vs con)** |
| --- | --- | --- |
| Up-regulated | 168 | 2.0013-6.5475 |
| Down-regulated | 470 | 0.0157-0.4999 |
| Total | 638 |  |
