# Supplementary material for: Overexpression of miR-155 in the Liver of Transgenic Mice Alters the Expression Profiling of Hepatic Genes Associated with Lipid Metabolism
Source: PLoS One. 2015 Mar 23;10(3):e0118417. doi: 10.1371/journal.pone.0118417 (PMC4370457; doi:10.1371/journal.pone.0118417)
Supplement: S5 Table — (DOC) [file pone.0118417.s009.doc]

**Table S5.** **Differentially expressed genes**

**involved in hepatic lipid, cholesterol and triacylglycerol metabolism between control and Rm155LG/Alb-Cre transgenic mice**

| **Differential expression** | **Number of genes** | **Fold difference (miR-19/con)** |
| --- | --- | --- |
| Up-regulated | 22 | 2.0548-4.2215 |
| Down-regulated | 67 | 0.0265-0.4968 |
| Total | 89 |  |
