# Supplementary material for: Overexpression of miR-155 in the Liver of Transgenic Mice Alters the Expression Profiling of Hepatic Genes Associated with Lipid Metabolism
Source: PLoS One. 2015 Mar 23;10(3):e0118417. doi: 10.1371/journal.pone.0118417 (PMC4370457; doi:10.1371/journal.pone.0118417)
Supplement: S6 Table — (DOC) [file pone.0118417.s010.doc]

**Table S6. Hepatic lipid metabolism-related genes differentially expressed between control and Rm155LG/Alb-Cre transgenic mice (average of three biological replicates >2 fold-change, t-test p < 0.05)**

| **Gene symbol** | **Description (Full name)** | **Fold difference**  **(155 vs con)** |
| --- | --- | --- |
|  | **PPAR signaling pathway** |  |
| Cyp4a14 | cytochrome P450, family 4, subfamily a, polypeptide 14 | 3.3415 |
| Acaa1b | acetyl-Coenzyme A acyltransferase 1B | 2.6937 |
| Fabp2 | fatty acid binding protein 2, intestinal | 2.2756 |
| Cyp4a10 | cytochrome P450, family 4, subfamily a, polypeptide 10 | 2.0571 |
| Pck1 | phosphoenolpyruvate carboxykinase 1, cytosolic | 2.0548 |
| Cyp7a1 | cytochrome P450, family 7, subfamily a, polypeptide 1 | 0.6171 |
| Acsl5 | acyl-CoA synthetase long-chain family member 5 | 0.4002 |
| Fabp4 | fatty acid binding protein 4, adipocyte | 0.3999 |
| Fads2 | fatty acid desaturase 2 | 0.3946 |
| Acsl3 | acyl-CoA synthetase long-chain family member 3 | 0.3807 |
| Cd36 | CD36 antigen | 0.3548 |
| Cyp8b1 | cytochrome P450, family 8, subfamily b, polypeptide 1 | 0.3241 |
| Lpl | lipoprotein lipase | 0.3097 |
| Fabp5 | fatty acid binding protein 5 | 0.1114 |
|  |  |  |
|  | **Adipocytokine signaling pathway** |  |
| Adipor2 | adiponectin receptor 2 | 2.1374 |
| Pck1 | phosphoenolpyruvate carboxykinase 1, cytosolic | 2.0548 |
| Adipor1 | adiponectin receptor 1 | 0.6356 |
| Acsl5 | acyl-CoA synthetase long-chain family member 5 | 0.4002 |
| Acsl3 | acyl-CoA synthetase long-chain family member 3 | 0.3807 |
| Cd36 | CD36 antigen | 0.3548 |
|  |  |  |
|  | **Fatty acid synthesis** |  |
| Acaa1b | acetyl-Coenzyme A acyltransferase 1B | 2.6937 |
| Elovl5 | ELOVL family member 5, elongation of long chain fatty acids (yeast) | 0.4656 |
| Fads2 | fatty acid desaturase 2 | 0.3946 |
| Degs1 | degenerative spermatocyte homolog 1 | 0.3598 |
| Fdft1 | farnesyl diphosphate farnesyl transferase 1 | 0.2913 |
| Sc4mol | sterol-C4-methyl oxidase-like | 0.2888 |
| Elovl6 | ELOVL family member 6, elongation of long chain fatty acids (yeast) | 0.2780 |
| Dgat2 | diacylglycerol O-acyltransferase 2 | 0.1731 |
| Fasn | fatty acid synthase | 0.1582 |
| Sc5d | sterol-C5-desaturase | 0.1526 |
| Acly | ATP citrate lyase | 0.0687 |
|  |  |  |
|  | **Fatty acid oxidation** |  |
| Adipor2 | adiponectin receptor 2 | 2.1374 |
| Dci | dodecenoyl-Coenzyme A delta isomerase (3,2 trans-enoyl-Coenyme A isomerase) | 2.0874 |
| Adipor1 | adiponectin receptor 1 | 0.6356 |
| Hadhb | hydroxyacyl-CoenzymeAdehydrogenase/3-ketoacyl-Coenzyme A thiolase/enoyl-Coenzyme A hydratase (trifunctional protein), beta subunit | 0.5337 |
| Hacl1 | 2-hydroxyacyl-CoA lyase 1 | 0.4728 |
| Pex7 | peroxisome biogenesis factor 7 | 0.3902 |
| Ucp2 | uncoupling protein 2 (mitochondrial, proton carrier) | 0.3607 |
|  |  |  |
|  | **Lipid transport** |  |
| Nolc1 | nucleolar and coiled-body phosphoprotein 1 | 4.2215 |
| Fabp2 | fatty acid binding protein 2, intestinal | 2.2756 |
| Fabp1 | fatty acid binding protein 1, liver | 1.8884 |
| Slc27a2 | solute carrier family 27 (fatty acid transporter), member 2 | 1.6889 |
| Cpt2 | carnitine palmitoyltransferase 2 | 1.6746 |
| Cpt1a | carnitine palmitoyltransferase 1a, liver | 1.6267 |
| Gm2a | GM2 ganglioside activator protein | 0.5204 |
| Slc27a5 | solute carrier family 27 (fatty acid transporter), member 5 | 0.5181 |
| Fabp4 | fatty acid binding protein 4, adipocyte | 0.3999 |
| Oprs1 | opioid receptor, sigma 1 | 0.1939 |
| Fabp5 | fatty acid binding protein 5, epidermal | 0.1114 |
|  |  |  |
|  | **Lipid storage** |  |
| DGAT2 | diacylglycerol O-acyltransferase 2 | 0.1731 |
|  |  |  |
|  | **Lipogenesis** |  |
| Cpt2 | carnitine palmitoyltransferase 2 | 1.6746 |
| Cpt1a | carnitine palmitoyltransferase 1a, liver | 1.6267 |
| Acaca | acetyl-Coenzyme A carboxylase alpha | 0.5571 |
| Srebf1 | sterol regulatory element binding factor 1 | 0.3045 |
| Fasn | fatty acid synthase | 0.1582 |
| Acly | ATP citrate lyase | 0.0687 |
|  |  |  |
|  | **VLDL export** |  |
| Slco1a1 | solute carrier organic anion transporter family, member 1a1 | 2.2066 |
| Ldlr | low density lipoprotein receptor | 0.5281 |
| Slco1a4 | solute carrier organic anion transporter family, member 1a4 | 0.4064 |
|  |  |  |
|  | **Fatty acid metabolism** |  |
| Cyp4a14 | cytochrome P450, family 4, subfamily a, polypeptide 14 | 3.3415 |
| Acaa1b | acetyl-Coenzyme A acyltransferase 1B | 2.6937 |
| Cyp7b1 | cytochrome P450, family 7, subfamily b, polypeptide 1 | 2.6268 |
| Peci | peroxisomal delta3, delta2-enoyl-Coenzyme A isomerase | 2.3338 |
| Adipor2 | adiponectin receptor 2 | 2.1374 |
| Dci | dodecenoyl-Coenzyme A delta isomerase | 2.0874 |
| Cyp4a10 | cytochrome P450, family 4, subfamily a, polypeptide 10 | 2.0571 |
| Pck1 | phosphoenolpyruvate carboxykinase 1, cytosolic | 2.0548 |
| Gba | glucosidase, beta, acid | 0.4899 |
| Hacl1 | 2-hydroxyacyl-CoA lyase 1 | 0.4728 |
| Pcsk9 | proprotein convertase subtilisin/kexin type 9 | 0.4650 |
| Scap | SREBF chaperone | 0.4417 |
| Hsd11b1 | hydroxysteroid 11-beta dehydrogenase 1 | 0.4406 |
| Asah1 | N-acylsphingosine amidohydrolase 1 | 0.4156 |
| Acsl5 | acyl-CoA synthetase long-chain family member 5 | 0.4002 |
| Fabp4 | fatty acid binding protein 4, adipocyte | 0.3999 |
| Hpgd | hydroxyprostaglandin dehydrogenase 15 (NAD) | 0.3869 |
| Hadh | hydroxyacyl-Coenzyme A dehydrogenase | 0.3866 |
| Acsl3 | acyl-CoA synthetase long-chain family member 3 | 0.3807 |
| Aldh9a1 | aldehyde dehydrogenase 9, subfamily A1 | 0.3752 |
| Insig1 | insulin induced gene 1 | 0.3622 |
| Pon1 | paraoxonase 1 | 0.3607 |
| Adh1 | alcohol dehydrogenase 1 (class I) | 0.3477 |
| Plcl2 | phospholipase C-like 2 | 0.3186 |
| Srebf1 | sterol regulatory element binding factor 1 | 0.3045 |
| Sult2a2 | sulfotransferasefamily2A, dehydroepiandrosterone(DHEA)-preferring, member 2 | 0.2726 |
| Fabp5 | fatty acid binding protein 5, epidermal | 0.1114 |
| Hao3 | hydroxyacid oxidase (glycolate oxidase) 3 | 0.0265 |
|  |  |  |
|  | **Lipid catabolism** |  |
| Lipg | lipase, endothelial | 3.8594 |
| Lpl | lipoprotein lipase | 0.3097 |
| Pnpla5 | patatin-like phospholipase domain containing 5 | 0.2168 |
|  |  |  |
|  | **LDL receptors and associated proteins** |  |
| Cxcl16 | chemokine (C-X-C motif) ligand 16 | 0.7332 |
| Ldlr | low density lipoprotein receptor | 0.5281 |
| Vldlr | very low density lipoprotein receptor | 0.5182 |
| Lrpap1 | low density lipoprotein receptor-related protein associated protein 1 | 0.4968 |
| Pcsk9 | proprotein convertase subtilisin/kexin type 9 | 0.4650 |
|  |  |  |
|  | **LDL associated proteins** |  |
| Scarf1 | scavenger receptor class F, member 1 | 2.2034 |
| Apoa4 | apolipoprotein A-IV | 0.4076 |
|  |  |  |
|  | **Fatty acid catabolism** |  |
| Nudt7 | nudix (nucleoside diphosphate linked moiety X)-type motif 7 | 3.1208 |
| Acox1 | acyl-Coenzyme A oxidase 1, palmitoyl | 1.5436 |
| Acad9 | acyl-Coenzyme A dehydrogenase family, member 9 | 0.4929 |
| Acsl5 | acyl-CoA synthetase long-chain family member 5 | 0.4002 |
| Acsl3 | acyl-CoA synthetase long-chain family member 3 | 0.3807 |
| Acss2 | acyl-CoA synthetase short-chain family member 2 | 0.1625 |
| Acly | ATP citrate lyase | 0.0687 |
|  |  |  |
|  | **Bile acid biosynthesis** |  |
| Acaa1b | acetyl-Coenzyme A acyltransferase 1B | 2.6937 |
| Cyp7a1 | cytochrome P450, family 7, subfamily a, polypeptide 1 | 0.6171 |
| Acad9 | acyl-Coenzyme A dehydrogenase family, member 9 | 0.4929 |
| Aldh9a1 | aldehyde dehydrogenase 9, subfamily A1 | 0.3752 |
| Adh1 | alcohol dehydrogenase 1 (class I) | 0.3477 |

**Note**: some genes related with hepatic lipid metabolism showing a fold change of more than 1.5 & less than 2 and a t test P value of less than 0.05 were also shown in this table.
