# Supplementary material for: Overexpression of miR-155 in the Liver of Transgenic Mice Alters the Expression Profiling of Hepatic Genes Associated with Lipid Metabolism
Source: PLoS One. 2015 Mar 23;10(3):e0118417. doi: 10.1371/journal.pone.0118417 (PMC4370457; doi:10.1371/journal.pone.0118417)
Supplement: S7 Table — (DOC) [file pone.0118417.s011.doc]

**Table S7. Hepatic cholesterol and triacylglycerol metabolism-related genes differentially expressed between control and Rm155LG/Alb-Cre transgenic mice (average of three biological replicates >2 fold-change, t-test p < 0.05)**

| **Gene symbol** | **Description (Full name)** | **Fold difference**  **(155 vs con)** |
| --- | --- | --- |
|  | **Cholesterol biosynthesis** |  |
| Cyp7b1 | cytochrome P450, family 7, subfamily b, polypeptide 1 [Source:MarkerSymbol;Acc:MGI:104978]" | 2.6268 |
| Cnbp | cellular nucleic acid binding protein | 0.6273 |
| Cyb5r3 | cytochrome b5 reductase 3 | 0.5876 |
| Dhcr24 | 24-dehydrocholesterol reductase | 0.5162 |
| Tm7sf2 | transmembrane 7 superfamily member 2 [Source:MarkerSymbol;Acc:MGI:1920416] | 0.5118 |
| Nsdhl | NAD(P) dependent steroid dehydrogenase-like | 0.3972 |
| Fdps | farnesyl diphosphate synthetase | 0.3698 |
| Mvk | mevalonate kinase | 0.2948 |
| Fdft1 | farnesyl diphosphate farnesyl transferase 1 | 0.2913 |
| Sc4mol | sterol-C4-methyl oxidase-like [Source:MarkerSymbol;Acc:MGI:1913484] | 0.2888 |
| Cyp51 | cytochrome P450, family 51 | 0.2853 |
| Mvd | mevalonate (diphospho) decarboxylase | 0.2595 |
| Sqle | Squalene epoxidase | 0.2218 |
| Hmgcr | 3-hydroxy-3-methylglutaryl-Coenzyme A reductase | 0.1894 |
| Sc5d | sterol-C5-desaturase (fungal ERG3, delta-5-desaturase) homolog | 0.1526 |
|  |  |  |
|  | **Cholesterol transport** |  |
| Stard3 | START domain containing 3 [Source:MarkerSymbol;Acc:MGI:1929618] | 1.9863 |
| Ldlr | low density lipoprotein receptor | 0.5281 |
| Apoa4 | apolipoprotein A-IV | 0.4076 |
| Cd36 | CD36 antigen | 0.3548 |
|  |  |  |
|  | **Cholesterol catabolism** |  |
| Scarf1 | scavenger receptor class F, member 1 [Source:MarkerSymbol;Acc:MGI:2449455]" | 2.2034 |
| Cyp46a1 | cytochrome P450, family 46, subfamily a, polypeptide 1 [Source:MarkerSymbol;Acc:MGI:1341877]" | 1.6933 |
| Cyp7a1 | cytochrome P450, family 7, subfamily a, polypeptide 1 [Source:MarkerSymbol;Acc:MGI:106091]" | 0.6171 |
|  |  |  |
|  | **Cholesterol homeostasis** |  |
| Lcat | lecithin cholesterol acyltransferase [Source:MarkerSymbol;Acc:MGI:96755] | 0.7974 |
| Ldlr | very low density lipoprotein receptor [Source:MarkerSymbol;Acc:MGI:98935] | 0.5182 |
| Pcsk9 | proprotein convertase subtilisin/kexin type 9 [Source:MarkerSymbol;Acc:MGI:2140260] | 0.4650 |
| Apoa4 | apolipoprotein A-IV | 0.4076 |
| Fabp4 | fatty acid binding protein 4, adipocyte [Source:MarkerSymbol;Acc:MGI:88038]" | 0.3999 |
|  |  |  |
|  | **Other genes involved in cholesterol metabolism** |  |
| Nr0b2 | nuclear receptor subfamily 0, group B, member 2 [Source:MarkerSymbol;Acc:MGI:1346344]" | 1.6789 |
| Osbpl1a | oxysterol binding protein-like 1A [Source:MarkerSymbol;Acc:MGI:1927551] | 0.6616 |
| Vldlr | very low density lipoprotein receptor [Source:MarkerSymbol;Acc:MGI:98935] | 0.5182 |
| Scap | SREBF chaperone  [Source:MarkerSymbol;Acc:MGI:2135958] | 0.4417 |
| Insig1 | insulin induced gene 1 | 0.3622 |
| Insig1 | insulin induced gene 1 | 0.3622 |
| Pon1 | paraoxonase 1 | 0.3607 |
| Srebf1 | sterol regulatory element binding factor 1 | 0.3045 |
|  |  |  |
|  | **Triglyceride synthesis** |  |
| Pcsk9 | proprotein convertase subtilisin/kexin type 9 [Source:MarkerSymbol;Acc:MGI:2140260] | 0.4650 |
| Ppap2c | phosphatidic acid phosphatase type 2c [Source:MarkerSymbol;Acc:MGI:1354945] | 0.4139 |
| Ces3 | carboxylesterase 3 | 0.2754 |
| Dgat2 | diacylglycerol  O-acyltransferase 2 [Source:MarkerSymbol;Acc:MGI:1915050] | 0.1731 |
| Ppap2a | phosphatidic acid phosphatase 2a  [Source:MarkerSymbol;Acc:MGI:108412] | 0.1439 |
|  |  |  |
|  | **Triacylglycerol catabolism** |  |
| Gk2 | serum/glucocorticoid regulated kinase 2 [Source:MarkerSymbol;Acc:MGI:1351318] | 0.4679 |
| Lpl | Lipoprotein lipase | 0.3097 |

**Note**: some genes related with cholesterol and triacylglycerol metabolism showing a fold change of more than 1.5 & less than 2 and a t test P value of less than 0.05 were also shown in this table.
