# Supplementary material for: Overexpression of miR-155 in the Liver of Transgenic Mice Alters the Expression Profiling of Hepatic Genes Associated with Lipid Metabolism
Source: PLoS One. 2015 Mar 23;10(3):e0118417. doi: 10.1371/journal.pone.0118417 (PMC4370457; doi:10.1371/journal.pone.0118417)
Supplement: S8 Table — (DOC) [file pone.0118417.s012.doc]

**Table S8. Gene ontology (GO) and KEGG pathway analysis of differentially expressed genes (related with hepatic lipid metabolism) from Rm155LG/Alb-Cre transgenic mice to control mice**

**GO terms representing biological process**

| **GO ID** | **GO terms representing biological process (GO description)** | **Count** | **Genes**  **(red = upregulated, green = downregulated)** | ***p*-value** |
| --- | --- | --- | --- | --- |
| GO:0006695 | cholesterol biosynthesis | 8 | **Hmgcr;Fdps;Mvk;Cyb5r3;Cyp51;Mvd;Nsdhl;Fdft1** | 1.26E-08 |
| GO:0006085 | acetyl-CoA biosynthesis | 2 | **Acss2;Acly** | 4.84E-06 |
| GO:0055089 | fatty acid homeostasis | 1 | **Got1** | 2.02E-04 |
| GO:0019915 | sequestering of lipid | 3 | **Gm2a;Hexb;Hexa;** | 3.44E-04 |
| GO:0006633 | fatty acid biosynthesis | 6 | **Sc4mol;Elovl5;Degs1;Sc5d;Fads2;Fasn** | 4.88E-04 |
| GO:0006646 | phosphatidylethanolamine biosynthesis | 1 | **Etnk2** | 6.01E-04 |
| GO:0006114 | glycerol biosynthesis | 1 | **Got1** | 6.01E-04 |
| GO:0008203 | cholesterol metabolism | 6 | **Pon1;Srebf1;Scap;Insig1;Cyp7b1;Pcsk9** | 0.002422 |
| GO:0006631 | fatty acid metabolism | 7 | **Fabp4;Dci;Hadh;Acsl5;Cyp4a10;Hpgd;Acsl3** | 0.003256 |
| GO:0006629 | lipid metabolism | 20 | **Asah1;Fabp5;Pon1;Sult2a2;Srebf1;Hsd11b1;Dci; Scap;Insig1;Cyp7b1;Adipor2;Gba;Hadh;Pck1;Pcsk9;Hacl1;Acsl5;Hpgd;Acsl3;Plcl2** | 0.004057 |
| GO:0006635 | fatty acid beta-oxidation | 2 | **Dci;Pex7** | 0.005223 |
| GO:0030497 | fatty acid elongation | 1 | **Elovl6** | 0.014224 |
| GO:0042632 | cholesterol homeostasis | 2 | **Fabp4;Pcsk9** | 0.01672 |
| GO:0050746 | regulation of lipoprotein metabolism | 1 | **Lipg** | 0.028246 |
| GO:0019217 | regulation of fatty acid metabolism | 1 | **Scap** | 0.029446 |
| GO:0001561 | fatty acid alpha-oxidation | 1 | **Hacl1** | 0.042069 |
| GO:0032803 | regulation of low-density lipoprotein receptor catabolic process | 1 | **Pcsk9** | 0.055695 |
| GO:0006651 | diacylglycerol biosynthesis | 1 | **Ppap2a** | 0.055695 |
| GO:0019432 | triacylglycerol biosynthesis | 1 | **Dgat2** | 0.082369 |
| GO:0006707 | cholesterol catabolism | 1 | **Cyp7a1** | 0.095423 |

**KEGG pathway analysis**

| **Pathway** | **Count** | **Genes**  **(red = upregulated, green = downregulated)** | ***p*-value** |
| --- | --- | --- | --- |
| PPAR signaling pathway | 13 | **Fabp5;Cd36;Fabp4;Cyp8b1;Lpl;Cyp7a1;Cyp4a14;Pck1;Acsl5;Cyp4a10;Fabp2;Acsl3;Fads2** | 4.03E-23 |
| Arachidonic acid metabolism | 10 | **Cyp2c38;Cyp2b9;Cyp2b10;Cyp2e1;Cyp4a14;Cyp2c40;Cyp2c50;Ggt6; Cyp4a10;Cyp4f13** | 5.56E-22 |
| Fatty acid metabolism | 10 | **Dci;Cyp4a14;Hadh;Acsl5;Adh1;Cyp4a10;Acaa1b;Acsl3;Peci;Aldh9a1** | 2.28E-17 |
| Biosynthesis of steroids | 9 | **Hmgcr;Sc4mol;Fdps;Mvk;Sc5d;Cyp51;Sqle;Nsdhl;Fdft1;** | 1.50E-15 |
| Linoleic acid metabolism | 6 | **Cyp3a11;Cyp3a44;Cyp2c38;Cyp2e1;Cyp2c50;Cyp2c40** | 2.98E-14 |
| Glycerolipid metabolism | 8 | **Lpl;Ppap2b;Akr1b7;Dgat2;Ppap2c; Ppap2a;Lipg;Aldh9a1** | 1.99E-09 |
| Biosynthesis of unsaturated fatty acids | 5 | **Elovl5;Elovl6;Acaa1b;Fads2;Fasn** | 4.55E-09 |
| Bile acid biosynthesis | 5 | **Acad9;Cyp7a1;Adh1;Acaa1b;Aldh9a1** | 4.55E-09 |
| Sphingolipid metabolism | 6 | **Asah1;Ppap2b; Degs1;Gba;Ppap2c;Ppap2a** | 5.99E-07 |
| Adipocytokine signaling pathway | 5 | **Cd36;Adipor2;Pck1;Acsl5;Acsl3** | 2.08E-05 |
| Glycerophospholipid metabolism | 4 | **Ppap2b;Pcyt2;Ppap2c;Ppap2a** | 1.14E-04 |
| Butanoate metabolism | 2 | **Hadh;Aldh9a1** | 2.25E-04 |
| Ether lipid metabolism | 3 | **Ppap2b;Ppap2c;Ppap2a** | 0.002368526 |
| Glycosphingolipid biosynthesis-globoseries | 2 | **Hexb;Hexa** | 0.005063788 |
| Glycosphingolipid biosynthesis-ganglioseries | 2 | **Hexb;Hexa** | 0.007663165 |
| Fatty acid biosynthesis | 1 | **Fasn** | 0.048857398 |
| Fatty acid elongation in mitochondria | 1 | **Hadh** | 0.080097994 |
| Glycosphingolipid biosynthesis-lactoseries | 1 | **St3gal4** | 0.080097994 |
