# Supplementary material for: Overexpression of miR-155 in the Liver of Transgenic Mice Alters the Expression Profiling of Hepatic Genes Associated with Lipid Metabolism
Source: PLoS One. 2015 Mar 23;10(3):e0118417. doi: 10.1371/journal.pone.0118417 (PMC4370457; doi:10.1371/journal.pone.0118417)
Supplement: S9 Table — (DOC) [file pone.0118417.s013.doc]

**Table S9. Vitamin, amino acid, nucleic acid, hormone metabolism- and hepatic drug metabolizing enzyme (DME)-related genes differentially expressed between control and Rm155LG/Alb-Cre transgenic mice (average of three biological replicates >2 fold-change, t-test p<0.05)**

| **Gene**  **symbol** | **Description (Full name)** | **Fold difference**  **(155 vs con)** |
| --- | --- | --- |
|  | **Retinol metabolism** |  |
| Retsat | retinol saturase (all trans retinol 13,14 reductase) | 4.2123 |
| Ugt2b1 | UDP glucuronosyltransferase 2 family, polypeptide B1 | 2.7765 |
| Cyp4a14 | cytochrome P450, family 4, subfamily a, polypeptide 14 | 3.3415 |
| Aldh1a1 | aldehyde dehydrogenase family 1, subfamily A1 | 2.2571 |
| Cyp4a10 | cytochrome P450, family 4, subfamily a, polypeptide 10 | 2.0571 |
| Rdh11 | retinol dehydrogenase 11 | 0.3821 |
| Adh1 | alcohol dehydrogenase 1 (class I) | 0.3477 |
| Cyp2c50 | cytochrome P450, family 2, subfamily c, polypeptide 50 | 0.3234 |
| Cyp2a12 | cytochrome P450, family 2, subfamily a, polypeptide 12 | 0.3032 |
| Cyp3a41 | cytochrome P450, family 3, subfamily a, polypeptide 41 | 0.2852 |
| Cyp2c38 | cytochrome P450, family 2, subfamily c, polypeptide 38 | 0.2680 |
| Cyp3a11 | cytochrome P450, family 3, subfamily a, polypeptide 11 | 0.2598 |
| Ugt1a6a | UDP glucuronosyltransferase 1 family, polypeptide A7C | 0.1882 |
| Dgat2 | diacylglycerol O-acyltransferase 2 | 0.1731 |
| Ugt3a2 | UDP glycosyltransferases 3 family, polypeptide A2 | 0.1713 |
| Ugt2b37 | UDP glucuronosyltransferase 2 family, polypeptide B37 | 0.1628 |
| Cyp2b10 | cytochrome P450, family 2, subfamily b, polypeptide 10 | 0.1257 |
| Cyp3a44 | cytochrome P450, family 3, subfamily a, polypeptide 44 | 0.1193 |
| Cyp2a5 | cytochrome P450, family 2, subfamily a, polypeptide 5 | 0.0814 |
| Cyp2b9 | cytochrome P450, family 2, subfamily b, polypeptide 9 | 0.0775 |
| Cyp2c40 | cytochrome P450, family 2, subfamily c, polypeptide 40 | 0.0500 |
|  |  |  |
|  | **Riboflavin metabolism** |  |
| Enpp3 | ectonucleotide pyrophosphatase/phosphodiesterase 3 | 2.7555 |
| Acp6 | acid phosphatase 6, lysophosphatidic | 0.3715 |
|  |  |  |
|  | **Drug metabolism - cytochrome P450** |  |
| Cyp2d9 | cytochrome P450, family 2, subfamily d, polypeptide 9 | 4.8180 |
| Gsta2 | glutathione S-transferase, alpha 2 (Yc2) | 3.3525 |
| Ugt2b1 | UDP glucuronosyltransferase 2 family, polypeptide B1 | 2.7765 |
| Gstp1 | glutathione S-transferase, pi 1 | 2.5690 |
| Gstp2 | Glutathione S-transferase P 2 (EC 2.5.1.18) (GST YF-YF) (GST-piA) (GST class-pi) (Gst P2) | 2.2637 |
| Cyp2e1 | cytochrome P450, family 2, subfamily e, polypeptide 1 | 2.1943 |
| Aox1 | aldehyde oxidase 1 | 2.1041 |
| Cyp2c39 | cytochrome P450, family 2, subfamily c, polypeptide 39 | 0.6780 |
| Adh1 | alcohol dehydrogenase 1 (class I) | 0.3477 |
| Cyp2c50 | cytochrome P450, family 2, subfamily c, polypeptide 50 | 0.3234 |
| Cyp2a12 | cytochrome P450, family 2, subfamily a, polypeptide 12 | 0.3032 |
| Cyp2c38 | cytochrome P450, family 2, subfamily c, polypeptide 38 | 0.2680 |
| Cyp3a11 | cytochrome P450, family 3, subfamily a, polypeptide 11 | 0.2598 |
| Ugt1a6a | UDP glucuronosyltransferase 1 family, polypeptide A7C | 0.1882 |
| Cyp2b10 | cytochrome P450, family 2, subfamily b, polypeptide 10 | 0.1257 |
| Cyp3a44 | cytochrome P450, family 3, subfamily a, polypeptide 44 | 0.1193 |
| Fmo3 | flavin containing monooxygenase 3 | 0.1032 |
| Cyp2a5 | cytochrome P450, family 2, subfamily a, polypeptide 5 | 0.0814 |
| Cyp2b9 | cytochrome P450, family 2, subfamily b, polypeptide 9 | 0.0775 |
| Cyp2c40 | cytochrome P450, family 2, subfamily c, polypeptide 40 | 0.0500 |
|  |  |  |
|  | **Metabolism of xenobiotics by cytochrome P450** |  |
| Gsta2 | glutathione S-transferase, alpha 2 (Yc2) | 3.3525 |
| Ugt2b1 | UDP glucuronosyltransferase 2 family, polypeptide B1 | 2.7765 |
| Gstp1 | glutathione S-transferase, pi 1 | 2.5690 |
| Gstp2 | Glutathione S-transferase P 2 (EC 2.5.1.18) (GST YF-YF) (GST-piA) (GST class-pi) (Gst P2) | 2.2637 |
| Cyp2e1 | cytochrome P450, family 2, subfamily e, polypeptide 1 | 2.1943 |
| Cyp2c39 | cytochrome P450, family 2, subfamily c, polypeptide 39 | 0.6780 |
| Adh1 | alcohol dehydrogenase 1 (class I) | 0.3477 |
| Cyp2c50 | cytochrome P450, family 2, subfamily c, polypeptide 50 | 0.3234 |
| Cyp2c38 | cytochrome P450, family 2, subfamily c, polypeptide 38 | 0.2680 |
| Cyp3a11 | cytochrome P450, family 3, subfamily a, polypeptide 11 | 0.2598 |
| Ugt1a6a | UDP glucuronosyltransferase 1 family, polypeptide A7C | 0.1882 |
| Cyp2f2 | cytochrome P450, family 2, subfamily f, polypeptide 2 | 0.1711 |
| Cyp2b10 | cytochrome P450, family 2, subfamily b, polypeptide 10 | 0.1257 |
| Cyp3a44 | cytochrome P450, family 3, subfamily a, polypeptide 44 | 0.1193 |
| Cyp2b9 | cytochrome P450, family 2, subfamily b, polypeptide 9 | 0.0775 |
| Cyp2c40 | cytochrome P450, family 2, subfamily c, polypeptide 40 | 0.0500 |
|  |  |  |
|  | **Drug metabolism - other enzymes** |  |
| Ugt2b1 | UDP glucuronosyltransferase 2 family, polypeptide B1 | 2.7765 |
| Tk1 | thymidine kinase 1 | 0.5491 |
| Upb1 | ureidopropionase, beta | 0.4827 |
| Dpyd | dihydropyrimidine dehydrogenase | 0.4267 |
| Upp2 | uridine phosphorylase 2 | 0.3960 |
| Es1 | esterase 1 | 0.3764 |
| Cyp2a12 | cytochrome P450, family 2, subfamily a, polypeptide 12 | 0.3032 |
| Cyp3a11 | cytochrome P450, family 3, subfamily a, polypeptide 11 | 0.2598 |
| Ces3 | carboxylesterase 3 | 0.2754 |
| Ugt1a6a | UDP glucuronosyltransferase 1 family, polypeptide A7C | 0.1882 |
| Cyp3a44 | cytochrome P450, family 3, subfamily a, polypeptide 44 | 0.1193 |
| Cyp2a5 | cytochrome P450, family 2, subfamily a, polypeptide 5 | 0.0814 |
|  |  |  |
|  | **Amino acid metabolism** |  |
| Gsta2 | glutathione S-transferase, alpha 2 (Yc2) | 3.3525 |
| Acaa1b | acetyl-Coenzyme A acyltransferase 1B | 2.6937 |
| Gstp1 | glutathione S-transferase, pi 1 | 2.569 |
| Gss | glutathione synthetase | 2.558 |
| Agxt | alanine-glyoxylate aminotransferase 2-like 1 | 2.3266 |
| Alas1 | aminolevulinic acid synthase 1 | 2.2678 |
| Gstp2 | Glutathione S-transferase P 2 (EC 2.5.1.18) (GST YF-YF) (GST-piA) (GST class-pi) (Gst P2). | 2.2637 |
| Aox1 | aldehyde oxidase 1 | 2.1041 |
| Kmo | kynurenine 3-monooxygenase (kynurenine 3-hydroxylase) | 2.0794 |
| Lars2 | leucyl-tRNA synthetase, mitochondrial | 2.0441 |
| Upb1 | ureidopropionase, beta | 0.4827 |
| Gldc | glycine decarboxylase | 0.4801 |
| Bckdhb | branched chain ketoacid dehydrogenase E1, beta polypeptide | 0.4754 |
| Pipox | pipecolic acid oxidase | 0.4641 |
| Hexb | hexosaminidase B | 0.4549 |
| Sars | seryl-aminoacyl-tRNA synthetase | 0.4528 |
| Afmid | arylformamidase | 0.4516 |
| Srp9 | signal recognition particle 9 | 0.451 |
| Prodh2 | proline dehydrogenase (oxidase) 2 | 0.4401 |
| Ddc | dopa decarboxylase | 0.4324 |
| Sephs2 | selenophosphate synthetase 2 | 0.4295 |
| Dpyd | dihydropyrimidine dehydrogenase | 0.4267 |
| Gcat | glycine C-acetyltransferase (2-amino-3-ketobutyrate-coenzyme A ligase) | 0.4175 |
| Gck | glucokinase | 0.4165 |
| Hadh | hydroxyacyl-Coenzyme A dehydrogenase | 0.3866 |
| Idh2 | isocitrate dehydrogenase 2 (NADP+), mitochondrial | 0.3768 |
| Aldh9a1 | aldehyde dehydrogenase 9, subfamily A1 | 0.3752 |
| Got1 | glutamate oxaloacetate transaminase 1, soluble | 0.3579 |
| Adh1 | alcohol dehydrogenase 1 (class I) | 0.3477 |
| Ldha | lactate dehydrogenase A | 0.3349 |
| Asl | argininosuccinate lyase | 0.3166 |
| Ggt6 | gamma-glutamyltransferase 6 | 0.2982 |
| Hexa | hexosaminidase A | 0.2813 |
| Dlst | dihydrolipoamide S-succinyltransferase (E2 component of 2-oxo-glutarate complex) | 0.2386 |
| Gstt3 | glutathione S-transferase, theta 3 | 0.2046 |
| Idh1 | isocitrate dehydrogenase 1 (NADP+), soluble | 0.1906 |
| Cyb5r3 | cytochrome b5 reductase 3 | 0.1802 |
| Acss2 | acyl-CoA synthetase short-chain family member 2 | 0.1625 |
| Ass1 | argininosuccinate synthetase 1 | 0.1374 |
| G6pdx | glucose-6-phosphate dehydrogenase X-linked | 0.1257 |
|  |  |  |
|  | **Nucleic acid metabolism** |  |
| Npr2 | natriuretic peptide receptor 2 | 2.8631 |
| Enpp3 | ectonucleotide pyrophosphatase/phosphodiesterase 3 | 2.7555 |
| Pde6g | phosphodiesterase 6G, cGMP-specific, rod, gamma | 2.661 |
| Upb1 | ureidopropionase, beta | 0.4827 |
| Dpyd | dihydropyrimidine dehydrogenase | 0.4267 |
| Upp2 | uridine phosphorylase 2 | 0.396 |
| Uox | urate oxidase | 0.3839 |
| Pde9a | phosphodiesterase 9A | 0.3611 |
| Pde5a | phosphodiesterase 5A, cGMP-specific | 0.3559 |
| Pold2 | polymerase (DNA directed), delta 2, regulatory subunit | 0.34 |
|  |  |  |
|  | **Hormone metabolism** |  |
| Hsd3b5 | hydroxy-delta-5-steroid dehydrogenase, 3 beta- and steroid delta-isomerase 5 | 6.5424 |
| Ugt2b1 | UDP glucuronosyltransferase 2 family, polypeptide B1 | 2.7765 |
| Hsd11b1 | hydroxysteroid 11-beta dehydrogenase 1 | 0.4406 |
| Hsd17b2 | hydroxysteroid (17-beta) dehydrogenase 2 | 0.3931 |
| Ugt1a6a | UDP glucuronosyltransferase 1 family, polypeptide A7C | 0.3799 |
| Akr1c18 | aldo-keto reductase family 1, member C18 | 0.2238 |
| Cyp17a1 | cytochrome P450, family 17, subfamily a, polypeptide 1 | 0.0924 |
|  |  |  |
|  | **Terpenoid biosynthesis** |  |
| Fdps | farnesyl diphosphate synthetase | 0.3698 |
| Fdft1 | farnesyl diphosphate farnesyl transferase 1 | 0.2913 |
| Sqle | squalene epoxidase | 0.2218 |

**Note**: some genes related with vitamin metabolism and hepatic drug metabolizing enzyme showing a fold change of more than 1.5 & less than 2 and a t test P value of less than 0.05 were also shown in this table.
