# Supplementary material for: Overexpression of miR-155 in the Liver of Transgenic Mice Alters the Expression Profiling of Hepatic Genes Associated with Lipid Metabolism
Source: PLoS One. 2015 Mar 23;10(3):e0118417. doi: 10.1371/journal.pone.0118417 (PMC4370457; doi:10.1371/journal.pone.0118417)
Supplement: S10 Table — (DOC) [file pone.0118417.s014.doc]

**Table S10. Gene ontology (GO) and KEGG pathway analysis of differentially expressed genes (related with metabolism of amino acids, nucleic acids, vitamins, drugs and hormones, etc) from Rm155LG/Alb-Cre transgenic mice to control mice**

**GO terms representing biological process**

| **GO ID** | **GO terms representing biological process**  **(GO description)** | **Count** | **Genes**  **(red = upregulated, green = downregulated)** | ***p*-value** |
| --- | --- | --- | --- | --- |
| GO:0006700 | C21-steroid hormone biosynthesis | 2 | **Hsd3b5;Cyp17a1** | 6.74E-07 |
| GO:0006749 | glutathione metabolism | 5 | **Gstp1;Gstt3;Idh1;Gstp2;G6pdx** | 9.29E-06 |
| GO:0042572 | retinol metabolism | 3 | **Adh1;Aldh1a1;Rdh11** | 5.73E-04 |
| GO:0006532 | aspartate biosynthesis | 1 | **Got1** | 6.01E-04 |
| GO:0006533 | aspartate catabolism | 1 | **Got1** | 6.01E-04 |
| GO:0019550 | glutamate catabolism to aspartate | 1 | **Got1** | 6.01E-04 |
| GO:0019551 | glutamate catabolism to 2-oxoglutarate | 1 | **Got1** | 6.01E-04 |
| GO:0046069 | cGMP catabolism | 1 | **Pde5a** | 2.02E-04 |
| GO:0042573 | retinoic acid metabolism | 3 | **Aldh8a1;Adh1;Aldh1a1** | 0.001398 |
| GO:0006214 | thymidine catabolism | 1 | **Dpyd** | 0.014224 |
| GO:0009166 | nucleotide catabolism | 1 | **Upp2** | 0.020479 |
| GO:0006544 | glycine metabolism | 1 | **Gldc** | 0.02386 |
| GO:0006562 | proline catabolism | 1 | **Prodh2** | 0.042069 |

**KEGG pathway analysis**

| **Pathway** | **Count** | **Genes**  **(red = upregulated, green = downregulated)** | ***p*-value** |
| --- | --- | --- | --- |
| Retinol metabolism | 19 | **Cyp3a11**;**Cyp3a44**;**Ugt2b1**;**Cyp2c38**;**Cyp2a12**;**Ugt1a6a**;**Cyp2b9**;**Cyp2b10**;**Cyp2a5**;**Cyp4a14**;**Cyp2c40**;**Cyp2c50**;**Adh1**;**Dgat2**;**Cyp4a10**; **Retsat**;**Aldh1a1** | 3.27E-50 |
| Drug metabolism - cytochrome P450 | 19 | **Cyp3a11**;**Cyp3a44**;**Ugt2b1**;**Gstp1**;**Cyp2c38**;**Cyp2a12**;**Ugt1a6a**;**Cyp2b9**;**Aox1**;**Cyp2b10**;**Cyp2e1**;**Cyp2a5**;**Gsta2**;**Cyp2c40**;**Cyp2c50**;**Adh1**;**Gstp2**;**Fmo3**;**Cyp2d9** | 3.36E-49 |
| Metabolism of xenobiotics by cytochrome P450 | 15 | **Cyp3a11**;**Cyp3a44**;**Ugt2b1**;**Gstp1**;**Cyp2c38**;**Ugt1a6a**;**Cyp2b9**;**Cyp2b10**;**Cyp2e1**;**Gsta2**;**Cyp2c40**;**Cyp2f2**;**Cyp2c50**;**Adh1**;**Cyp2b10** | 1.07E-41 |
| Drug metabolism - other enzymes | 11 | **Cyp3a11;Cyp3a44;Ugt2b1;Ces3;Cyp2a12;Ugt1a6a;Cyp2a5;Dpyd;Upp2; Upb1;Es1** | 7.52E-34 |
| Androgen and estrogen metabolism | 5 | **Ugt2b1; Hsd11b1; Ugt1a6a; Hsd3b5;Hsd17b2** | 1.07E-21 |
| Glutathione metabolism | 8 | **Gstp1;Idh2;Gsta2;Gss;Ggt6;Idh1;Gstp2;G6pdx** | 1.68E-11 |
| C21-Steroid hormone metabolism | 4 | **Hsd11b1;Hsd3b5;Cyp17a1;Akr1c18** | 5.24E-10 |
| Glycine, serine and threonine metabolism | 6 | **Gcat;Pipox;Gldc;Sars;Agxt;Alas1** | 9.11E-08 |
| Valine, leucine and isoleucine degradation | 5 | **Bckdhb;Aox1;Hadh;Acaa1b;Aldh9a1** | 7.74E-08 |
| Tryptophan metabolism | 6 | **Aox1;Ddc;Hadh;Afmid;Kmo;Aldh9a1** | 6.56E-08 |
| Purine metabolism | 7 | **Pde5a;Uox;Pold2;Npr2;Enpp3;Pde9a;Pde6g** | 6.46E-06 |
| Terpenoid biosynthesis | 3 | **Sqle;Fdps;Fdft1** | 5.64E-06 |
| Aminosugars metabolism | 4 | **Cyb5r3;Gck;Hexb;Hexa** | 5.54E-06 |
| Lysine degradation | 4 | **Pipox;Hadh;Dlst;Aldh9a1** | 3.27E-06 |
| Protein export | 1 | **Srp9** | 2.22E-06 |
| Tyrosine metabolism | 4 | **Got1;Aox1;Ddc;Adh1** | 1.78E-05 |
| Pyrimidine metabolism | 4 | **Pold2;Dpyd;Upp2;Upb1** | 1.66E-05 |
| Selenoamino acid metabolism | 2 | **Sephs2;Ggt6** | 7.82E-04 |
| beta-Alanine metabolism | 3 | **Dpyd;Upb1;Aldh9a1** | 7.82E-04 |
| Phenylalanine metabolism | 2 | **Got1;Ddc** | 6.80E-04 |
| Riboflavin metabolism | 2 | **Enpp3; Acp6** | 2.95E-04 |
| Alanine and aspartate metabolism | 4 | **Got1;Agxt;Asl;Ass1** | 1.80E-04 |
| Arginine and proline metabolism | 4 | **Got1;Prodh2;Asl;Ass1** | 1.80E-04 |
| Propanoate metabolism | 4 | **Ldha;Acss2;Ldha;Aldh9a1** | 1.24E-04 |
| Valine, leucine and isoleucine biosynthesis | 1 | **Lars2** | 0.00238916 |
| Cysteine metabolism | 1 | **Ldha** | 0.005063788 |
| Histidine metabolism | 2 | **Ddc;Aldh9a1** | 0.021107261 |
| Glutamate metabolism | 2 | **Got1;Gss** | 0.027361292 |
